# Microrobotic Swarms for Cancer Therapy

**DOI:** 10.34133/research.0686

**Published:** 2025-04-29

**Authors:** Leiming Xie, Jinbo Liu, Zhen Yang, Hui Chen, Yibin Wang, Xingzhou Du, Yongping Fu, Peng Song, Jiangfan Yu

**Affiliations:** ^1^ Shenzhen Institute of Artificial Intelligence and Robotics for Society (AIRS), Shenzhen 518129, China.; ^2^ School of Science and Engineering, The Chinese University of Hong Kong, Shenzhen, Shenzhen 518172, China.; ^3^ Department of Cardiovascular Medicine, Affiliated Hospital of Shaoxing University, Shaoxing 312000, China.; ^4^ Department of Interventional Therapy, National Cancer Center/National Clinical Research Center for Cancer/Cancer Hospital and Shenzhen Hospital, Chinese Academy of Medical Sciences and Peking Union Medical College, Shenzhen 518116, China.

## Abstract

Microrobotic swarms hold great promise for the revolution of cancer treatment. The coordination of miniaturized microrobots offers a unique approach to treating cancers at the cellular level with enhanced delivery efficiency and environmental adaptability. Prior studies have summarized the design, functionalization, and biomedical applications of microrobotic swarms. The strategies for actuation and motion control of swarms have also been introduced. In this review, we first give a detailed introduction to microrobot swarming. We then explore the design of microrobots and microrobotic swarms specifically engineered for cancer therapy, with a focus on tumor targeting, infiltration, and therapeutic efficacy. Moreover, the latest developments in active delivery methods and imaging techniques that enhance the precision of these systems are discussed. Finally, we categorize and analyze the various cancer therapies facilitated by functional microrobotic swarms, highlighting their potential to revolutionize treatment strategies for different cancer types.

## Introduction

Microrobotic swarms are formed by more than hundreds of agents ranging from several nanometers to hundreds of micrometers, which can transform the energy from chemical fuels [1–3], biological fuels [4–6], or external physical fields, such as magnetic fields [7–10], electrical fields [11–14], acoustic fields [15–17], and optical fields [18–20], into motion, enabling active mobility in complex environments. Microrobotic swarms can actively deliver cargo to target sites within the body, minimizing systemic side effects [21–23]. Meanwhile, the swarms can transport therapeutic agents, such as drugs and cells [24–26], to treat diseases. Owing to the tiny size of swarm agents, the swarms can navigate through obstacles and narrow spaces, such as blood vessels [27–29] and tortuous lumens [30–32], performing shape reconfiguration. Swarms with a high concentration can enhance imaging contrast under different medical imaging modalities, enabling efficient diagnosis and assessment of disease states [33,34]. The unique advantages of microrobotic swarms provide exciting possibilities for their applications in complex physiological environments.

Cancer remains one of the most challenging diseases to treat [35], as conventional therapies like chemotherapy and radiotherapy are often hindered by poor specificity, systemic toxicity, and damage to healthy tissues [36]. In contrast, microrobotic swarms have emerged as a transformative approach, providing enhanced targeting precision, multimodal therapy, and minimally invasive capabilities. Unlike conventional methods that depend on passive diffusion or systemic circulation, microrobotic swarms actively navigate complex biological environments to deliver therapeutic agents directly to tumor sites, reducing off-target effects and allowing for real-time monitoring [37–39]. These swarms can perform multiple functions simultaneously, such as drug delivery [40], imaging [41], and hyperthermia [42], while adapting to dynamic environments for precise cancer treatment.

The functionalization [43], control [44], and biomedical applications [45] of individual microrobots have been reviewed. Microrobotic swarms provide distinct advantages over single-robot systems. Wang et al*.* [46] provided a comprehensive review of synthetic swarm systems, highlighting advances in swarm design and control. Law et al*.* [47] reviewed the fundamental principles and applications of microrobotic swarms. Chen et al*.* [48] summarized the diverse biomedical applications of microrobotic swarms, emphasizing their potential in targeted drug delivery and medical imaging. However, a timely and comprehensive review of the microrobotic swarms tailored for cancer therapy is still lacking. The design strategies of microrobotic agents for approaching tumor tissues across different organs are summarized in this review, considering the anatomical and pathological barriers inherent to various tumor environments.

**Fig. 1.**
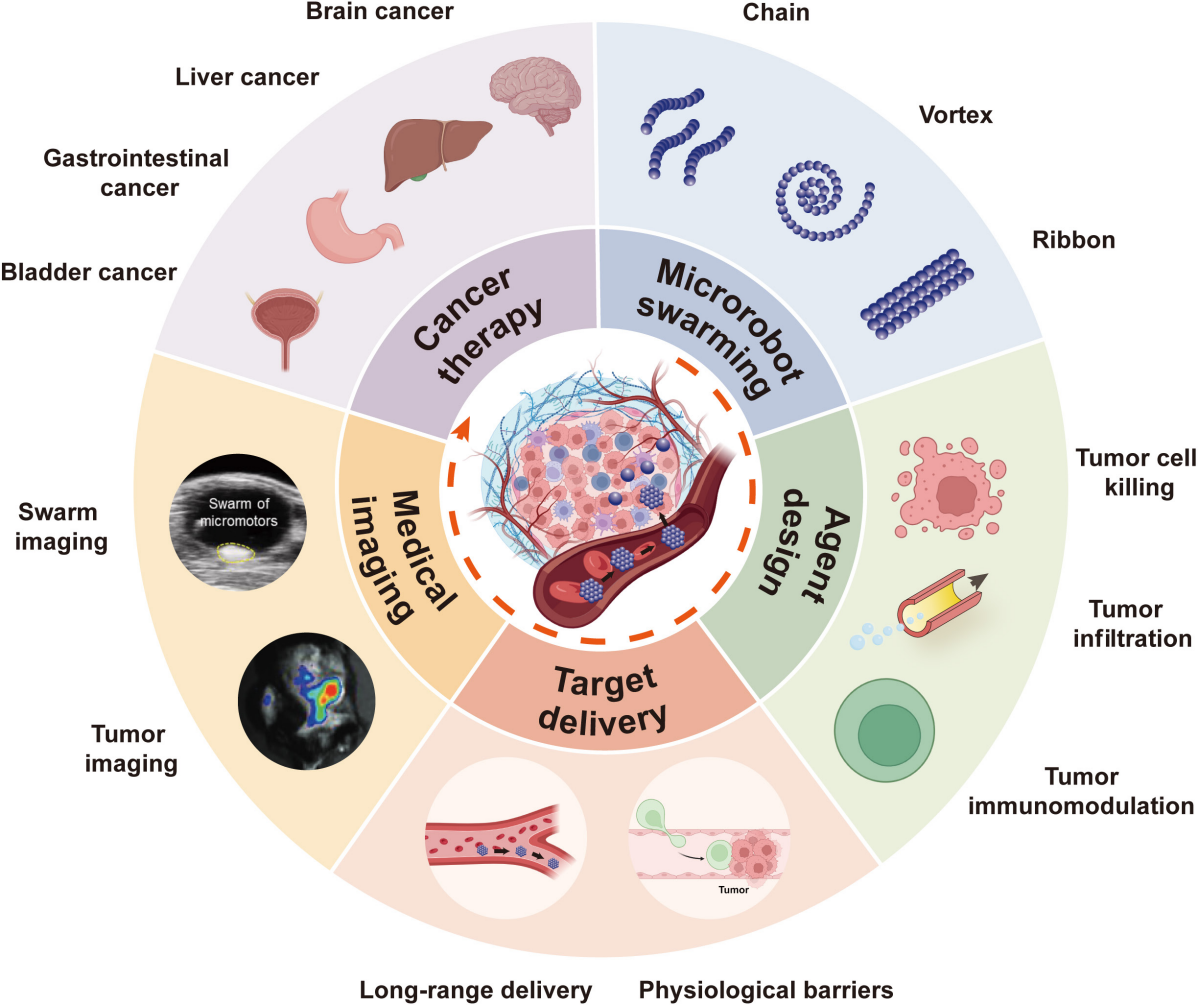
Schematic illustration of 5 aspects of microrobotic swarms in cancer therapy (microrobot swarming, agent design, targeted delivery, medical imaging, cancer therapy). The inserts are reproduced from the following references. Swarm imaging [143]. Copyright 2021, Wiley-VCH GmbH. Tumor imaging [195]. Copyright 2023, Wiley-VCH GmbH.

In this review, we summarize the applications of microrobots in cancer therapy from the perspective of swarms (Fig. 1). The microrobot swarming under different actuation fields is introduced. We then discuss the design of agents for cancer therapy from 3 aspects, that is, tumor cell elimination, tumor infiltration, and tumor immunomodulation. Thereafter, the delivery and imaging strategies of swarms in vivo are introduced. We further summarize the treatment of tumors in the brain, lung, liver, gastrointestinal (GI) tract, and bladder using swarms. Finally, the challenges and future directions to enhance cancer treatment efficiency are discussed.

## Microrobot Swarming

Swarms are decentralized, self-organized collectives of agents that interact locally with one another and their environment to exhibit coordinated global behaviors. Unlike natural swarms observed in biological systems, microrobot swarming is driven by externally applied fields, such as magnetic [49–53], electric [14,54–56], optical [57–60], or acoustic fields [61–64] or chemical fuels [65–68]. These engineered strategies enable the swarm to collectively navigate in complex environments, adapt to dynamic conditions, and effectively target tumor sites. Microrobot swarming refers to the coordinated collective behavior of microscale or nanoscale robots that self-organize through local interactions or external guidance to perform tasks requiring emergent functionalities. This review examines how the coordinated movement of microrobots is achieved through engineered actuation and control strategies, with a focus on their applications in precise tumor infiltration and enhanced therapeutic efficacy.

### Magnetic fields

**Fig. 2.**
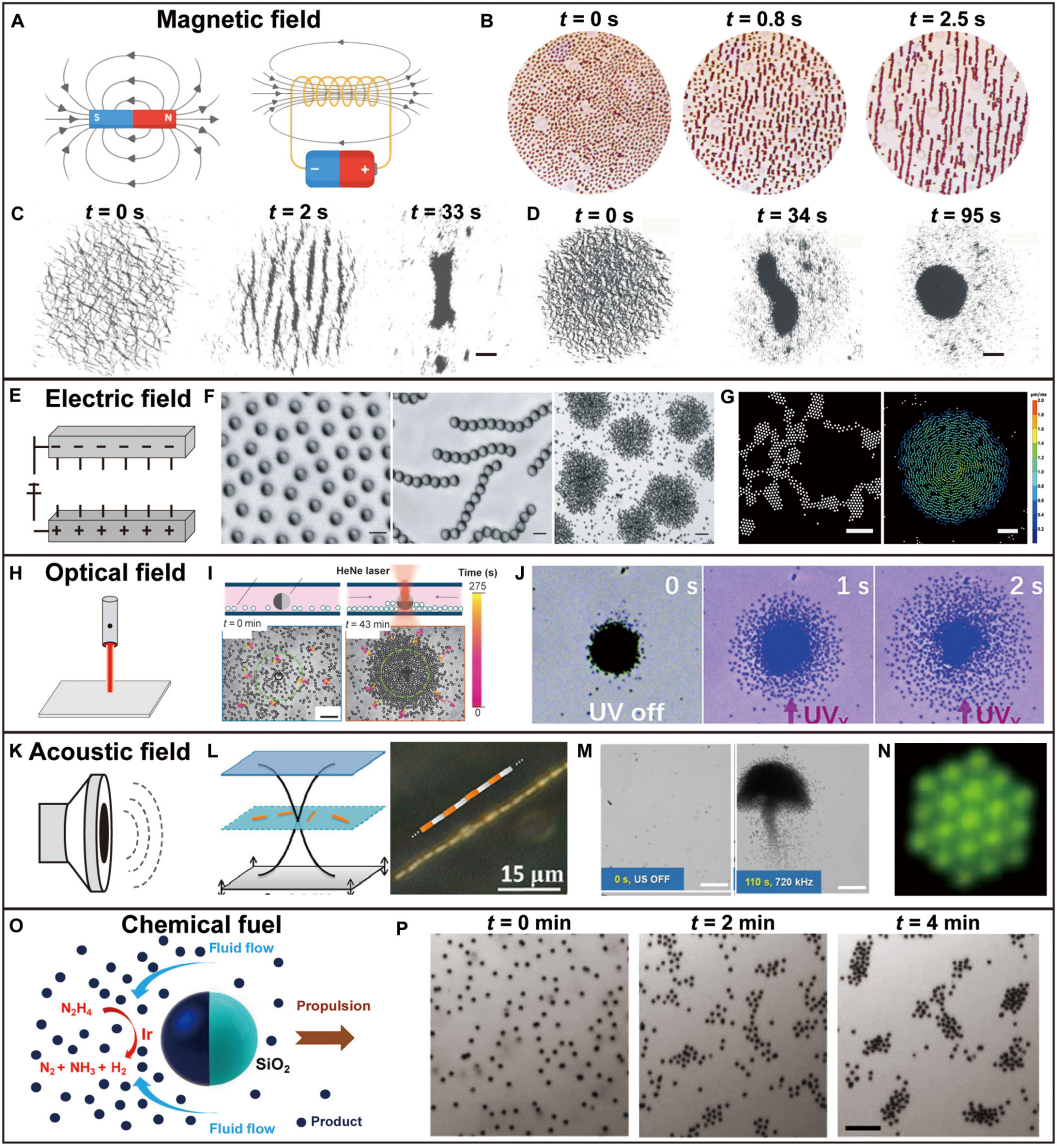
External field and chemical fuels guided microrobotic swarms. (A) Schematics illustrating the magnetic field generated by permanent magnets and solenoids with current. (B) Generation of a chain-like swarm under a magnetic field [73]. Copyright 2019, American Association for the Advancement of Science. (C) Generation of a ribbonlike swarm guided by an oscillating magnetic field [74]. Copyright 2018, Springer Nature. (D) Formation of a magnetic nanoparticle cluster with vortex-like behavior directed by a 2D rotating in-plane magnetic field [75]. Copyright 2018, SAGE. (E) Schematic illustrating an electric field. (F) Rearrangement of the swarm of Janus particles controlled by AC electric fields [76]. Copyright 2016, Springer Nature. (G) Formation of patterns through the collective movement of spherical particles [78]. Copyright 2021, National Academy of Sciences. (H) Schematic illustrating the optical field. (I) Reversible clustering of photoactivated colloidal particles near a carbon-coated Janus particle under UV light [80]. Copyright 2022, Springer Nature. (J) Pattern reconfiguration of TiO_2_ motors under UV light [81]. Copyright 2017, Wiley-VCH GmbH. (K) Schematic illustrating the acoustic field. (L) Arrangement of Au and AuRu rods into patterns at the nodal plane of a standing ultrasound wave [84]. Copyright 2012, American Chemical Society. (M) Generation of a dandelion-like nanorod swarm guided by an acoustic field [84]. Copyright 2020, by Wiley-VCH GmbH. (N) Organization of colloidal particles into patterns directed by multidimensional acoustic waves [63]. Copyright 2022, Springer Nature. (O) Schematic illustrating chemical-guided micro/nanorobotic swarms [65]. Copyright 2014, American Chemical Society. (P) Collective motion of Janus particles composed of catalytic Ir/SiO_2_ within the hydrazine-based fuel [65]. Copyright 2014, American Chemical Society.

Actuated by magnetic fields, magnetic materials experience magnetic torques and forces, showcasing different collective behaviors by programming field parameters, such as intensity, frequency, and orientation (Fig. 2A). Magnetic fields are widely used to actuate small-scale swarms due to their deep tissue penetration and high precision. By embedding magnetic materials in microrobots, external magnetic fields can generate both translational forces and rotational torques, which are governed by the magnetic dipole interactions [69]. Recent studies have focused on optimizing field configurations to enhance the targeting accuracy of the swarm [70–72]. When subjected to alternating magnetic fields, hematite colloidal particles interact with each other through dipole–dipole forces, transitioning from a dispersed, liquid-like state to a chain-like swarm (Fig. 2B) [73]. Additionally, Yu et al*.* [74] assembled paramagnetic isotropic nanoparticles (NPs) into ribbon-like swarms under oscillating magnetic fields (Fig. 2C). Besides, Yu et al*.* [75] investigated vortex-like swarms composed of Fe_3_O_4_ NPs by applying rotating magnetic fields (RMFs) (Fig. 2D).

### Electric fields

By tuning the input of electric fields, researchers can manipulate polar agents to form different swarms (Fig. 2E). Electric fields drive microrobots to move via electrophoretic or dielectrophoretic forces. When subjected to an electric field, microrobots undergo electrically induced polarization, generating gradient forces through dielectrophoresis [76]. The actuation process is governed by electrokinetic phenomena and can be modeled using theories such as Maxwell–Wagner polarization, which predicts particle behavior in nonuniform electric fields [77]. This approach facilitates rapid response times and high control precision. Electrostatic interactions induced by alternating current (AC) electric fields can induce asymmetric interactions between metal-dielectric Janus colloids and lead to different swarm behaviors. The swarm behaviors of the particles are mainly affected by the ion screening effect and the dipole interaction between the particles. At low frequencies (∼kHz), the ion screening effect dominates, weakening the dipole interactions between the particles, and the particles exhibit a gas-like swarm. At moderate frequencies (20 to 50 kHz), the ion screening effect weakens, and the dipole interactions guide the particles to form clusters. At high frequencies (MHz), the ion screening effect becomes negligible, and the particles form chains due to the dipole interactions. Adding salt to the solution increases the ion concentration, strengthening the dipole interactions between the particles and leading to the aggregation of Janus particles into clusters (Fig. 2F) [76]. Dielectric colloidal particles acquire antiparallel electric dipoles under uniform vertical electric fields. As the amplitude of the electric field increases and exceeds the threshold value, the particles begin to rotate and eventually form vortex-like swarms (Fig. 2G) [78].

### Optical fields

Optical fields are capable of inducing diverse swarm behaviors in specific materials and functionalized micro/nanoagents (Fig. 2H). Optical fields enable actuation through light-induced forces and photothermal effects. Photon transfer momentum to the microrobotic swarms can be actuated by directly transferring momentum through photons or by the flow induced through the local heating effects [79]. Although offering high spatial resolution, optical actuation is limited by penetration depth, which demands proper designs in clinical applications. Optical fields can activate the self-propulsion of colloidal particles and particle–particle attractive interactions. Competition between the 2 appears to lead to self-assembly behaviors (Fig. 2I) [80]. Moreover, Mou et al*.* [81] investigated that hydroxyl-group-enriched TiO_2_ motors can aggregate into clusters through electrolyte diffusiophoresis. Laser light causes the overlap of the asymmetric electrolyte cloud around particles, activating nonelectrolyte diffusiophoretic and promoting the swelling behavior (Fig. 2J).

### Acoustic fields

Researchers manipulate microrobots by tuning the amplitude modulation and frequency of the acoustic field (Fig. 2K). Acoustic fields utilize ultrasonic waves to generate motion through acoustic radiation forces. Gradient forces generated by the pressure difference can drive the microrobotic swarms to perform linear and rotational movements [82]. The mechanism involves the balance between acoustic radiation forces and viscous drag forces. When a standing wave is established, microrobots experience a net force proportional to the acoustic pressure gradient and acoustic contrast factor. The gradient force drives the swarms toward pressure nodes or antinodes, while viscous drag forces oppose the motion [83]. The pressure gradient created by nodes and antinodes in the acoustic field is used to drive particles. Metal nanorods aggregate along the nodal lines to form chains under the combination of vertical standing waves and van der Waals forces (Fig. 2L) [84]. Li et al*.* [85] proposed an ultrasound-driven Ga–In alloy liquid metal colloidal motor. Under a 720-kHz standing wave acoustic field, the Ga–In colloidal motors first aggregate to form stripe-shaped swarms and then transform into aggregation (Fig. 2M). By applying Fourier synthesis of harmonics, researchers can synthesize sound waves to autonomously assemble particles into diverse structures, such as 3-dimensional (3D) clusters, 2D crystals, and 1D chains (Fig. 2N) [63].

### Chemical fuel

In addition to the external fields mentioned above, chemical fuel is also an important driving source for the motion of microrobots, with osmotic gradients being a primary mechanism (Fig. 2O) [65]. Chemical fields utilize reaction–diffusion processes to create self-propelling forces in microrobots [86]. The actuation mechanism includes diffusiophoresis, where concentration gradients generate movement, and chemotactic responses, enabling autonomous movement that adapts to specific chemical triggers through reaction–diffusion models and nonequilibrium thermodynamics [87]. Microrobotic swarms can be synthesized with chemotaxis for short-range navigation to reach target sites. Silicon particles with an iridium hemispherical coating catalyze the decomposition of hydrazine into nitrogen gas, hydrogen gas, and ammonia. The decomposition products accumulate around the iridium surface, and the osmotic gradient causes water to flow from regions of low solute concentration to high concentration. As a result, the Janus particles are pushed toward the silicon dioxide side. An inward fluid flux is thus created, which facilitates the formation of small swarms, continually attracting nearby particles, eventually leading to the formation of cluster swarms (Fig. 2P) [65].

Under the guidance of different external fields, microrobots can achieve the generation and reconfiguration of swarm patterns [88]. The fundamental actuation mechanisms that form microrobot swarms include the utilization of magnetic, acoustic, optical, electric, and chemical fields. A theoretical framework has been covered for a deeper understanding of the swarm dynamics in complex environments, paving the way for the design of agents in cancer therapy.

## Design of Agents for Cancer Therapy

The functions of microrobotic swarms mainly rely on the properties of agents. Based on tumor characteristics, the design of swarm agents for cancer therapy can be summarized from 3 major aspects: tumor cell targeting and killing, tumor infiltration, and cancer immunomodulation. Meanwhile, all agents have the capability of active motion, allowing them to penetrate and accumulate within tumor tissues.

### Agents for tumor cell targeting and killing

**Fig. 3.**
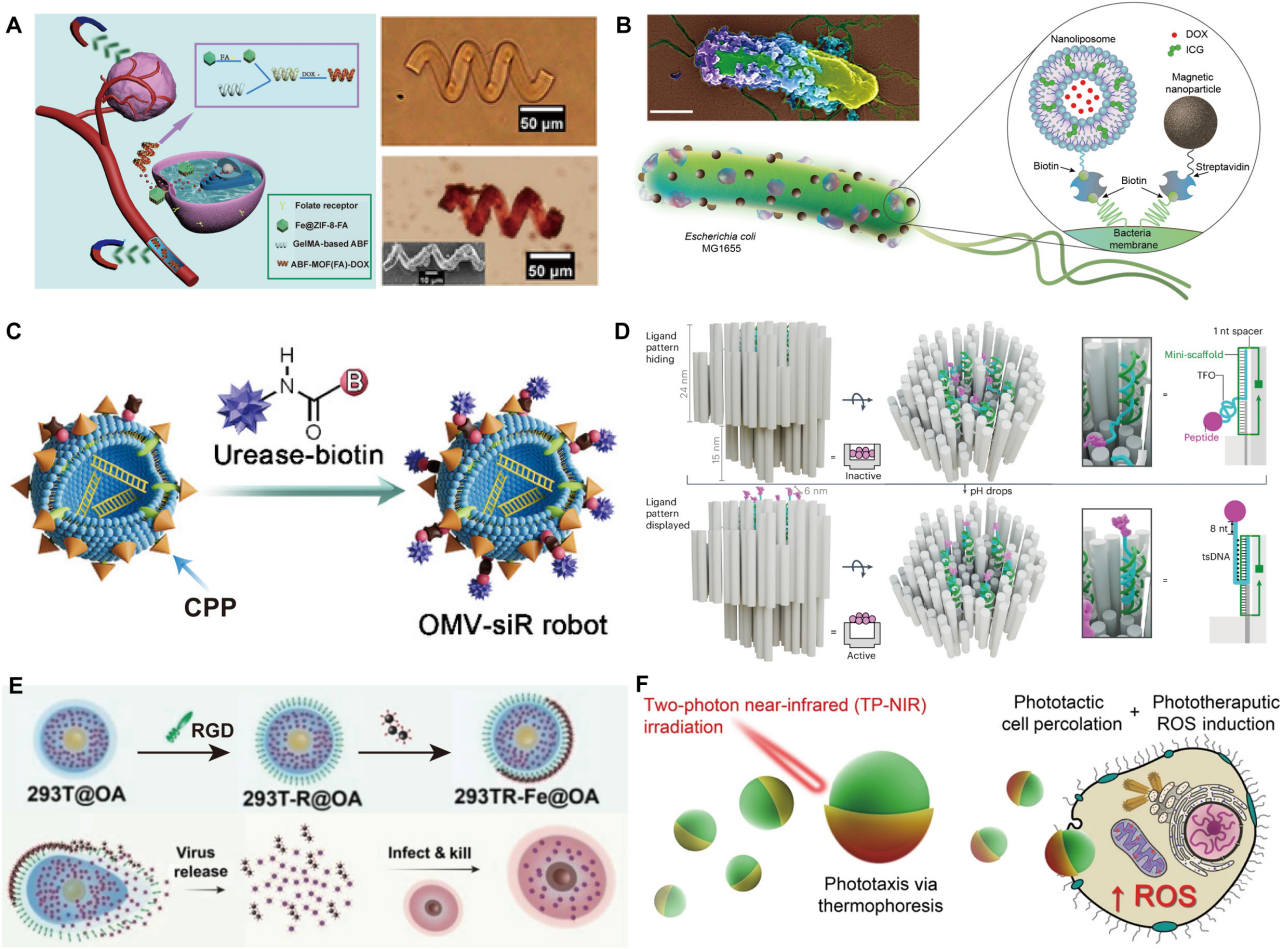
Design of agents for tumor cell killing. (A) Depiction of artificial bacterial flagellar (ABF) microrobots, maneuvered through magnetic control, for cancer treatment targeting folate receptors [93]. Copyright 2023, Beijing Institute of Technology Press. (B) Diagram depicting biohybrid microrobots derived from bacteria, integrated with magnetic nanoparticles and nanoliposomes [4]. Copyright 2022, American Association for the Advancement of Science. (C) Fabrication process of OMV-siR robots with urease modification [104]. Copyright 2024, PNAS. (D) pH-responsive origami switch design for ligand concealment and presentation [107]. Copyright 2024, Springer Nature. (E) Schematic illustrating the fabrication process of cell robots loaded with OA [8]. Copyright 2022, Wiley-VCH GmbH. (F) Upon TP-NIR stimulation, nanomotors exhibit 2 simultaneous actions: boosting cellular engagement and internalization (through percolation) while producing ROS for precise, localized cytotoxic effects [19]. Copyright 2021, Springer Nature.

Tumor cells can proliferate indefinitely [89]. Currently, chemotherapy is the general treatment method for tumors. However, systemic chemotherapy has poor tumor targeting, causing harm to normal tissues. Tumor-targeted molecules are used to modify the surface of microrobots. Folic acid (FA) is a small molecule ligand with a high biological affinity that can specifically recognize folate receptors [90,91]. The folate receptor reductase on the surface of cancer cells can enable the magnetic microrobots carrying FA to enter the cell, allowing the drug to enter the cytoplasm, and enhancing the therapeutic effect [92]. Ye et al. [93] developed magnetically driven microrobots composed of methacryloyl gelatin (GelMA) and a porous magnetic metal–organic framework, effectively loading substantial amounts of anticancer drugs doxorubicin (DOX) and FA (Fig. 3A). Under magnetic field guidance, microrobots can efficiently localize areas around lesions.

Chemotherapy drugs can be loaded on organisms with autonomous motility and tumor-targeting capabilities to improve drug delivery efficiency [6,37,38,94]. Akolpoglu et al. [4] introduced a biohybrid microrobotic platform utilizing genetically engineered motile bacteria, which can be fabricated in a batch (Fig. 3B). Stimuli-responsive delivery of active cargo is realized by developing a liposome that encapsulates drug molecules, such as DOX, and photothermal agents like indocyanine green. Bacterial biohybrids activated by near-infrared (NIR) light enable the on-demand release of anticancer drugs. The innovative approach enhances the precision of drug delivery and minimizes the systemic side effects commonly associated with traditional chemotherapy. Furthermore, the stimuli-responsive mechanisms allow for controlled release of therapeutic agents, providing the opportunity for tailored treatment regimes.

Gene therapy for tumors has seen substantial growth in recent years [95]. For gene therapy of tumor cells, small interfering RNA (siRNA) is a type of nucleic acid drug that targets and silences the expression of target genes at the posttranscriptional level through the RNA interference mechanism [96,97]. However, unmodified naked siRNAs have a short half-life in the bloodstream and are easily cleared by the glomeruli [98]. Lipid NPs are widely used for siRNA delivery due to their ability to shield enclosed siRNA from nuclease degradation and elimination through the kidneys, transporting siRNA to target tissues and cells [99–102]. Outer membrane vesicles (OMVs) are a natural lipid body derived from bacteria [103]. Tang et al*.* [104] developed urea-powered OMV robots, which were functionalized with cell-penetrating peptides (CPPs) to achieve targeting and tumor penetration (Fig. 3C). Simultaneously, the contained siRNA is protected. Besides, DNA nanotechnology revolutionizes tasks such as molecule detection [105] and transportation [106] at the individual molecule level. Wang et al. [107] designed a 3D DNA origami switch targeting the tumor necrosis factor receptor superfamily, which can sense the weak acid environment of tumor tissue and display a cytotoxic ligand pattern, thereby activating the apoptotic mechanism of cancer cells (Fig. 3D).

Oncolytic virotherapy, which uses engineered oncolytic viruses (OVs), has become a promising cancer treatment approach [108]. The OV replicates within infected cells and takes control of the transcriptional machinery, eventually causing cell death and lysis after exhausting the host resources [109]. Meanwhile, new viruses are released, which continue to infect nearby tumor cells. However, the capsid of OV is easily recognized as a pathogen by the innate immune system, leading to its rapid elimination. Loading OV into natural cells (stem cells, tumor cells, macrophages) helps evade immune recognition due to the intrinsic nature of host cells [110–113], thereby preventing the clearance of OV. However, the lack of cancer specificity results in low delivery efficiency. By anchoring magnetic NPs to cell surfaces, Cong et al*.* [8] presented magnetically driven OV-loaded Janus cell microrobots (Fig. 3E). When actuated by external RMFs, the microrobots roll toward tumor sites in the bladder, enabling tumor-specific delivery of OV.

Phototherapy has emerged as a predominant modality in tumor treatment, encompassing photothermal therapy (PTT) and photodynamic therapy (PDT) [114]. When performing PTT, photothermal agents are irradiated with light of a particular wavelength, heating local tumor areas and eliminating tumor cells. In PDT, specific light irradiation is used to activate photosensitizers, generating a substantial quantity of reactive oxygen species (ROS) that selectively eliminate tumor cells. Cao et al*.* [19] introduced phototactic and phototherapeutic nanomotors comprising biodegradable block copolymers functionalized with aggregation-induced emission (AIE) motifs (Fig. 3F). A nanomotor is a nanoscale device capable of converting energy into mechanical motion, enabling it to perform tasks such as propulsion, cargo transport, or targeted drug delivery. The nanomotors efficiently convert 2-photon NIR radiation into motion, enhancing thermophoretic motility powered by an asymmetric gold nanoshell. Meanwhile, ROS is generated for phototherapeutic applications.

### Agents for characteristics of TME to promote tumor infiltration

The tumor microenvironment (TME) is a complex and dynamic ecosystem surrounding tumor cells, comprising different cells, extracellular matrix (ECM) components, and signaling molecules, which collectively influence cancer progression and response to therapy [115]. Understanding TME is crucial for developing targeted therapy methods, disrupting tumor-supportive processes, and enhancing antitumor immunity.

**Fig. 4.**
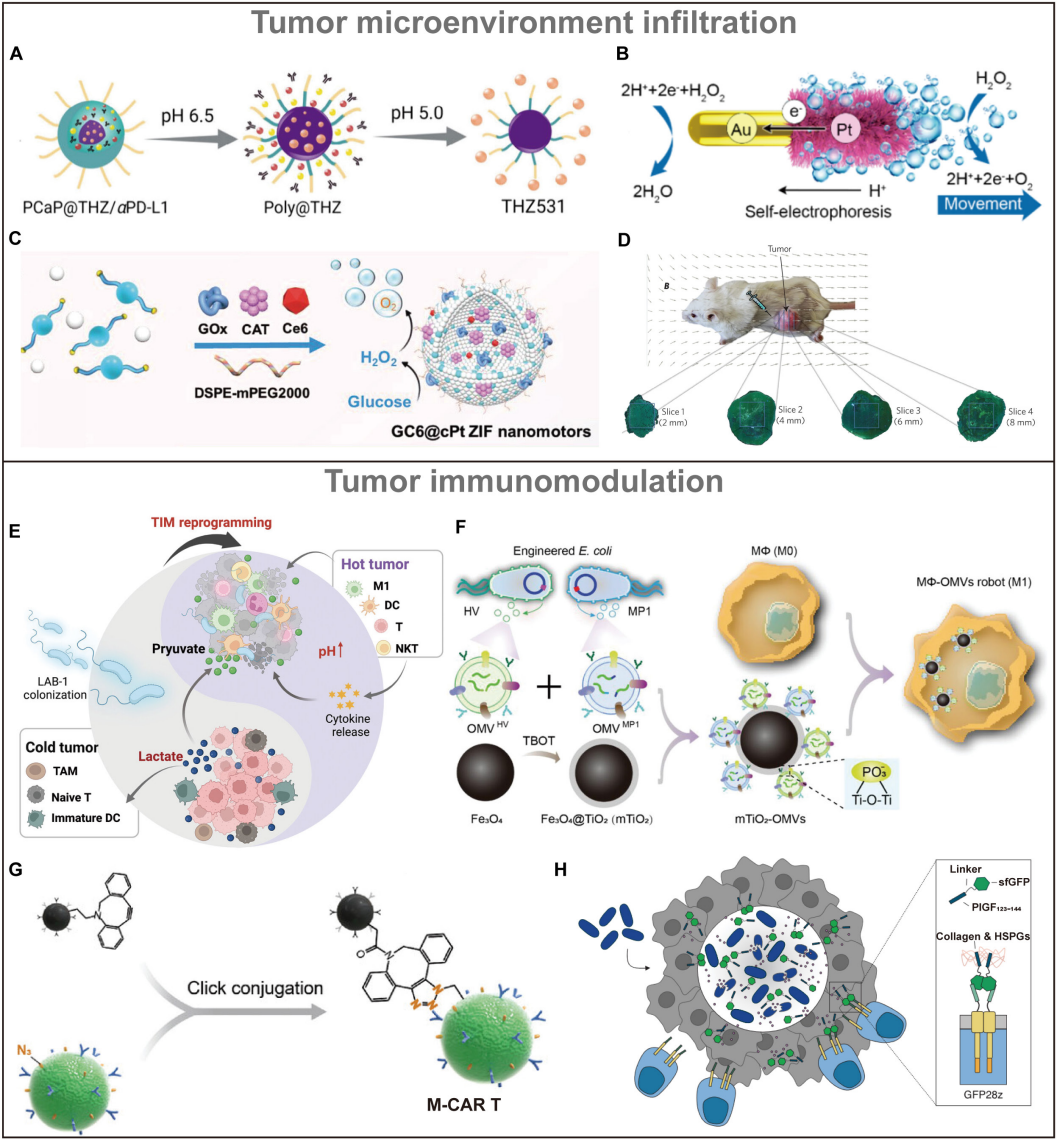
Design of agents for TME infiltration and immunomodulation. (A) Preparation of hybrid nanomotor PCaP@THZ/αPD-L1 [118]. Copyright 2024, American Chemical Society. (B) Schematic illustration of the synthesis route of the JAuNR-Pt nanomotor [122]. Copyright 2022, American Chemical Society. (C) Cisplatin-based ZIF nanomotors for encapsulation and dual-responsive release of GOx, CAT, and Ce6 [126]. Copyright 2023, Wiley-VCH GmbH. (D) Peritumoral injection of MC-1 into tumor xenografts in mice, along with representative tumor sections at various depths [130]. Copyright 2016, Springer Nature. (E) Schematic diagram of LAB-1 reprogramming the TIM [136]. Copyright 2024, Wiley-VCH GmbH. (F) Schematic of the fabrication of MΦ-OMVs robots [143]. Copyright 2023, Wiley-VCH GmbH. (G) Functionalized M-CAR Ts were successfully produced by conjugating N_3_-CAR T cells with dibenzocyclooctyne (DBCO)-modified beads using click chemistry [148]. Copyright 2023 Wiley-VCH GmbH. (H) A diagram illustrating the ProCAR system, where tumor-colonizing probiotic bacteria generate and secrete synthetic CAR targets (Tags) in situ, marking universal solid tumor elements for targeted destruction by GFP-CARs (GFP28z) [149]. Copyright 2023, American Association for the Advancement of Science.

Due to tumor cells not fully oxidizing glucose [116], the glycolysis pathway in tumor cells generates significant amounts of lactic acid, leading to a slightly acidic environment ranging from 6.5 to 6.8 [117]. The mildly acidic condition offers opportunities for developing microrobots. Xu et al. [118] developed acidity-activated polymer/calcium phosphate (CaP) hybrid nanomotors (Fig. 4A). In the acidic TME, the dissolution of the CaP layer not only releases αPD-L1 but also generates thermal energy for nanomotor locomotion, enhancing the cellular uptake of therapeutic drugs and enabling deep penetration into tumors.

Hydrogen peroxide (H_2_O_2_) plays a pivotal role as a ROS within tumors, functioning as an important signaling agent in diverse physiological processes such as cellular growth, proliferation, and the aging process [119,120]. Compared to normal tissues, the concentration of H_2_O_2_ is typically higher in tumors [121]. This characteristic offers several strategies for tumor treatment: On one hand, endogenous H_2_O_2_ in the tumor can be catalytically decomposed to generate oxygen (O_2_) in situ, addressing the issue of hypoxia; on the other hand, nanomaterials containing H_2_O_2_-specific chemical bonds can enable TME-responsive therapies [2]. Li et al*.* [122] reported a Janus gold nanorod-platinum (JAuNR-Pt) microrobot (Fig. 4B). The electrons transfer from Pt nanoshells to Au nanorods in the presence of H_2_O_2_, resulting in rapid autonomous motion to increase tumor infiltration. Meanwhile, enzymes, as naturally occurring biological catalysts, facilitate the transformation of substrates into products. Catalase (CAT), glucose oxidase (GOx), and urease have been effectively utilized in the creation of enzyme-powered microrobots, enabling autonomous movement through enzymatic reactions under mild conditions [3,123–125]. Yu et al*.* [126] developed microrobot encapsulation of GOx, CAT, and Ce6 using cisplatin-skeletal zeolitic imidazolate frameworks (cPt ZIFs) (Fig. 4C). Overproduced H_2_O_2_ was decomposed by CAT to generate O_2_ to relieve hypoxia.

Low oxygen levels (hypoxia) are common in tumors due to irregular blood supply and rapid tumor cell proliferation [127]. Hypoxia can activate the pathways that promote tumor survival, angiogenesis (formation of new blood vessels), and metastasis [128]. Concurrently facilitating optimal amassed proliferation environments for specific bacteria inclined toward hypoxic metabolism [37,40]. Magnetotactic bacteria are employed as a responsive model organism to magnetic fields [129]. Felfoul et al*.* [130] successfully developed a microrobot based on magnetotactic bacteria. Guided by an external magnetic field and the bacterial ability to sense low-oxygen regions, these microrobots can efficiently transport therapeutic agents to deep tumor sites. As Fig. 4D shows, the bacteria were injected around the tumor and traced with fluorescently labeled bacteria (green). It was found that the magnetic field can make the bacteria penetrate deep into the tumor and spread throughout the tumor.

### Agents for tumor immunomodulation

Immunotherapies have transformed our approach to diseases like cancer by harnessing the body’s natural defenses to target and eliminate diseased cells [131]. Tumor immunotherapy is a cancer treatment strategy by utilizing the body’s immune system to recognize and eliminate cancer cells. Tumor immunotherapy has great potential as it works by activating the body’s immune system to kill tumor cells [132]. However, the immune response is often suppressed at the tumor site, and it has become a major obstacle to the effectiveness of immunotherapy [133]. Tumor cells disguise themselves as normal cells very effectively so that immune cells fail to detect any abnormalities [134]. This type of tumor with low immune infiltration is called the cold tumor [135]. In cold tumors, lactate protects tumors from immune attack and promotes their progression by suppressing the immune activity of various immune cells. Regarding this feature, Ma et al*.* [136] identified a novel high-lactate-metabolizing photosynthetic bacterium called LAB-1, which can reprogram the tumor immune microenvironment (TIM) and enhance immunotherapy efficacy by reducing lactate levels and stimulate immune cell response (Fig. 4E). Bacteria possess inherent tumor-targeting capabilities, and their surface components or metabolic byproducts can stimulate the immune system, inducing antitumor responses [137], such as peptidoglycan and lipopolysaccharide (LPS) [138,139]. Besides, bacterial OMVs are naturally produced by the membrane of Gram-negative bacteria during growth and have recently become immunotherapeutic agents for a variety of biomedical applications [140,141]. OMVs can convert M2 macrophages into M1 macrophages [142]. Based on these capabilities, Li et al*.* [143] developed a magnetically driven macrophage microrobot loaded with OMVs that express antitumor peptides (Fig. 4F). Combining the tumor-inhibitory effects of M1 macrophages with the immunostimulatory properties of OMVs can significantly enhance the body’s antitumor immunity.

Additionally, chimeric antigen receptor T (CAR-T) cell therapy is a significant type of immunotherapy that has demonstrated considerable potential in treating hematological malignancies [144,145]. However, its effectiveness in solid tumors has been limited due to challenging physical barriers and immunosuppressive microenvironments [146,147]. Tang et al. [148] used click chemistry to modify immunomagnetic beads onto the surface of CAR-T cells, based on the magnetic acoustic sequential drive. (Fig. 4G). Another approach is to improve tumor targeting of CAR-T cells. Vincent et al*.* [149] developed a platform where probiotic bacteria guide CAR-T cells to target hard-to-reach tumors by releasing synthetic CAR targets, enhancing the effectiveness of CAR-T cell therapy (Fig. 4H). This approach is divided into 2 steps. The first step involves modifying a nonpathogenic *Escherichia coli* strain to deliver synthetic antigens to the TME and “mark” the tumor; the second step is to generate CAR-T cells that can recognize these synthetic antigen markers. When the *E. coli* probiotic is applied, CAR-T cells can attack solid tumors in a targeted manner.

The design of microrobotic agent lays the foundation for their functional capabilities. With the purpose of active delivery of therapeutic payloads to tumor sites, investigations into active navigation and key barriers to overcome will be reviewed.

## Delivery of Swarm in Cancer Therapy

Traditional nanomedicines primarily reach tumor sites through passive diffusion and accumulate in tumor tissues via the enhanced permeability and retention (EPR) effect [150]. Certain macromolecules of specific sizes, such as liposomes, NPs, and some large molecular drugs, tend to penetrate tumor tissues more easily and remain there longer compared to normal tissues [151]. However, increasing evidence indicates that only about 0.7% of administered NPs typically reach solid tumors [152,153]. Researchers have pointed out for the first time that there is a dense basement membrane structure on the outside of tumor blood vessels, which severely hinders the ability of nanomedicines to permeate beyond the vessels, resulting in a “vascular pooling” accumulation of nanomedicines outside the tumor vasculature [154]. These limitations significantly restrict the clinical effectiveness of current nanocarriers and explain the scarcity of approved nanomedicines for cancer treatment to date. Microrobotic swarms integrate the benefits of conventional nanomedicines, including drug protection, selectivity, and biocompatibility, while also enabling active movement. This presents significant potential for improving both long-range and short-range targeted treatment of tumors. Here, we discuss 3 methods for long-range delivery and the main physiological barriers for short-range delivery.

### Long-range delivery

Long-range delivery of microrobotic swarms requires optimal delivery routes based on tumor location. Long-range delivery is the ability of microrobotic swarms to navigate through complex biological environments from the point of administration (e.g., superficial intravenous injection) to the target tumor site while maintaining their structural integrity and functional efficacy. For instance, the delivery of drugs to GI tumors bypasses the need for systemic circulation to reach the target lesion, thereby circumventing physiological filtration processes, which occur in the liver and kidneys [155,156]. In contrast, for tumors located in more challenging sites, such as brain cancer, it is essential to enhance drug accumulation at the tumor site while minimizing systemic clearance. In this section, we have outlined 3 methodologies for effective long-range drug delivery.

#### Real-time guidance

**Fig. 5.**
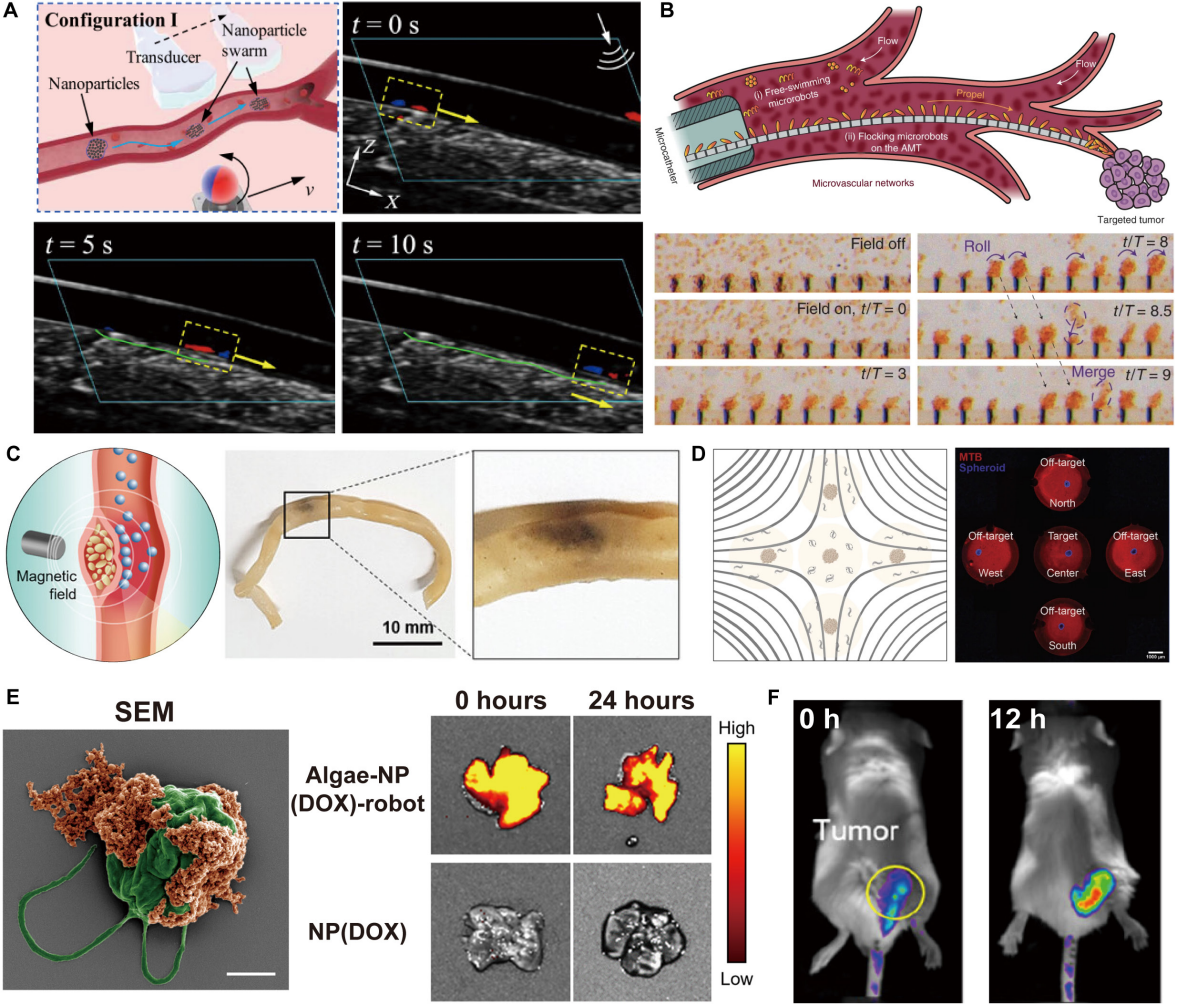
(A) Schematic of swarm navigation in blood vessels. Navigation of the microswarm under ultrasound Doppler guidance [159]. Copyright 2021, American Association for the Advancement of Science. (B) Graphical illustration of AMT deployment in microvascular networks. Images showing the organization and coordinated motion of microparticles at specific time intervals [161]. Copyright 2021, Springer Nature. (C) MAP@IO microparticles are directed by external magnetic fields and accumulate at the designated cancer site in the GI tract, promoting extended retention in the local region [7]. Copyright 2021, Wiley-VCH GmbH. (D) Schematic of the experimental setup. Blue spheroids are surrounded by the red MTB dispersion, which has been tagged with a NIR proliferation dye to allow for the tracking of daughter cells as they proliferate [162]. Copyright 2024, Springer Nature. (E) Pseudo-colored scanning electron microscope (SEM) image of an algae-NP (DOX)-robot, with algae depicted in green and NP(DOX) in orange. The lung distribution of microalgae was assessed using ex vivo fluorescence imaging at multiple time intervals following the intratracheal administration of the algae-NP (DOX)-robot or NP(DOX) [164]. Copyright 2024, American Association for the Advancement of Science. (F) Fluorescent images of mice with tumors were captured at 0- and 12-h intervals following the administration of microrobots along with the applied magnetic field [165]. Copyright 2021, American Chemical Society.

Real-time guidance of microrobotic swarms in cancer therapy allows for precise intervention. By leveraging advanced imaging technologies such as positron emission tomography (PET) [157] and magnetic resonance imaging (MRI) [41,158], swarms can navigate through complex biological environments, delivering therapeutic agents precisely to malignant tissues. Wang et al. [159] developed a method for real-time navigation of magnetic NP swarms using ultrasound Doppler imaging to facilitate active endovascular delivery. As illustrated in Fig. 5A, ultrasound Doppler imaging captures the swarm’s downward movement over time under the influence of a magnetic field. This approach enhances the precision of targeted delivery and enables continuous real-time monitoring of the swarms’ trajectory.

In current minimally invasive therapies, medical catheters stand out as a stable and reliable delivery solution, allowing drugs to reach specific locations [160]. However, at small scales, fluidic viscous forces become notably significant when using a closed transmission system. To overcome this obstacle, Gu et al*.* [161] have devised a magnetic artificial microtube (AMT), embedding numerous small magnets within a solid fiber, to enable the rapid and effective transport of magnetic microrobotic swarms within tortuous blood vessels. As Fig. 5B shows, under an RMF, the microparticles initially assembled in a swarm and then propelled collectively along the AMT. In comparison to freely moving microrobots, these swarms transported along the AMT exhibit increased speed and robustness.

#### Potential well for swarm delivery

The concept of the potential well is designed to capture microrobotic swarms within systemic circulation. By creating a localized region of enhanced attraction, this potential well can effectively retain and guide microrobots as they navigate through the bloodstream. The capability allows for sustained interaction with specific tissues or tumors, improving the precision and efficacy of treatment while minimizing off-target effects. Choi et al. [7] introduced magnetically controllable drug carriers designed to firmly bind to target locations using a bioengineered mussel adhesive protein for localized therapeutic delivery in the turbulent esophageal environment (Fig. 5C). The strategy serves as a magnetically steerable mechanism to load, transport, and release drugs at specific sites over extended durations. Magnetic guidance and sustained retention were evaluated ex vivo in a rat small intestine under flow conditions. The particles demonstrated prolonged stability under magnetic influence, with optical imaging used to visualize their localization within the intestinal wall. Besides, Mirkhani et al*.* [162] explored a method for applying spatially specific torque density to microrobots by merging rotating fields with magnetostatic selection fields (Fig. 5D). Using magnetotactic bacteria as torque-based actuators, their numerical modeling demonstrated effective off-target torque suppression, suggesting potential for centimeter to millimeter resolution in human applications. With the addition of a selection field, colonization of the targeted spheroids by magnetotactic bacteria (MTB) remained largely unchanged, while a notable reduction in accumulation was seen at off-target locations under the superimposed selection field.

#### Autonomous delivery of swarms

Autonomous delivery is a method in which microrobotic swarms can navigate into tumors independently, without the need for external physical stimulation. For instance, biomimetic microrobots inspired by biological systems draw their operational energy and movement from the natural actuation abilities of living microorganisms. These devices are designed to carry artificial payloads, enabling them to perform a wide range of functions, such as bacteria [130], algae [94], and sperm [163]. Magnetotactic bacteria can not only move under the control of a magnetic field but also spontaneously move to anaerobic areas. Sperm is also designed as a carrier for drug delivery due to its efficient autonomous movement. Meanwhile, algae exhibit self-sustained motion in confined environments, display phototactic behavior, emit autofluorescence for imaging, and adapt to diverse conditions. Zhang et al. [164] designed biohybrid microrobots using microalgae for targeted delivery of chemotherapeutic agents to treat lung metastases from melanoma. These algae-based systems enable autonomous movement within the lungs, facilitating controlled drug release and improved distribution to achieve antimetastatic outcomes. As shown in Fig. 5E, the distribution of microalgae in the lungs was assessed using ex vivo fluorescence imaging at different time points following the intratracheal administration of either algae-NP (DOX)-robot or NP(DOX). Notably, only the algae-NP(DOX)-robot group displayed a strong signal indicative of chloroplast autofluorescence. This finding reflects the improved accumulation and extended retention of the drug payload NP(DOX) within the lungs. Besides, certain endogenous cells, like macrophages (MΦ) and tumor cells [39,143], possess the capacity to detect chemotactic signals and navigate toward tumors. This inherent ability presents a promising avenue for modifying these cells to transport therapeutic compounds for tumor treatment. Nguyen et al*.* [165] pioneered the development of dual-targeting MΦ-based microrobots, leveraging the inherent tumor-homing proficiency of macrophages in conjunction with external magnetic guidance for anticancer interventions. Illustratively depicted in Fig. 5F, post-intravenous administration of the MΦ microrobot swarms, a gradual rise in fluorescence signal was observed within 12 h, underscoring the directional migration of macrophages and microrobots toward the tumor site.

Effective tumor targeting by microrobotic swarms requires a strategy to combine long-range delivery with adaptive responses to local biological barriers. Initially, long-range navigation is achieved through external actuation fields to propel the swarms from their injection site to the vicinity of the tumor. The microrobots will then encounter several challenges in entering tumor tissue, including irregular vascular permeability, dense ECM, and active immune responses. Recent efforts have focused on modifying the surface properties of microrobots and integrating stimuli-responsive payload release mechanisms to tackle the issue.

### Short-range delivery across pathological barriers

Short-range delivery refers to the localized transport of therapeutic agents from vascular compartments directly into tumor cells. When microrobotic swarms reach the tumor region, localized delivery of therapeutics in cancer therapy encounters several significant barriers that impede effective treatment outcomes. High interstitial pressure and a dense ECM hinder the penetration of therapeutic agents [166]. Additionally, the heterogeneity of tumors, marked by blood vessel abnormality, complicates consistent drug delivery, often leading to ineffective targeting of all cancer cells [167]. The thick basement membrane further restricts the ability of drugs to extravasate into tumor tissues. Together, these factors create a complex landscape that requires innovative strategies for improving the localized delivery of cancer therapeutics.

#### Crossing epithelial

**Fig. 6.**
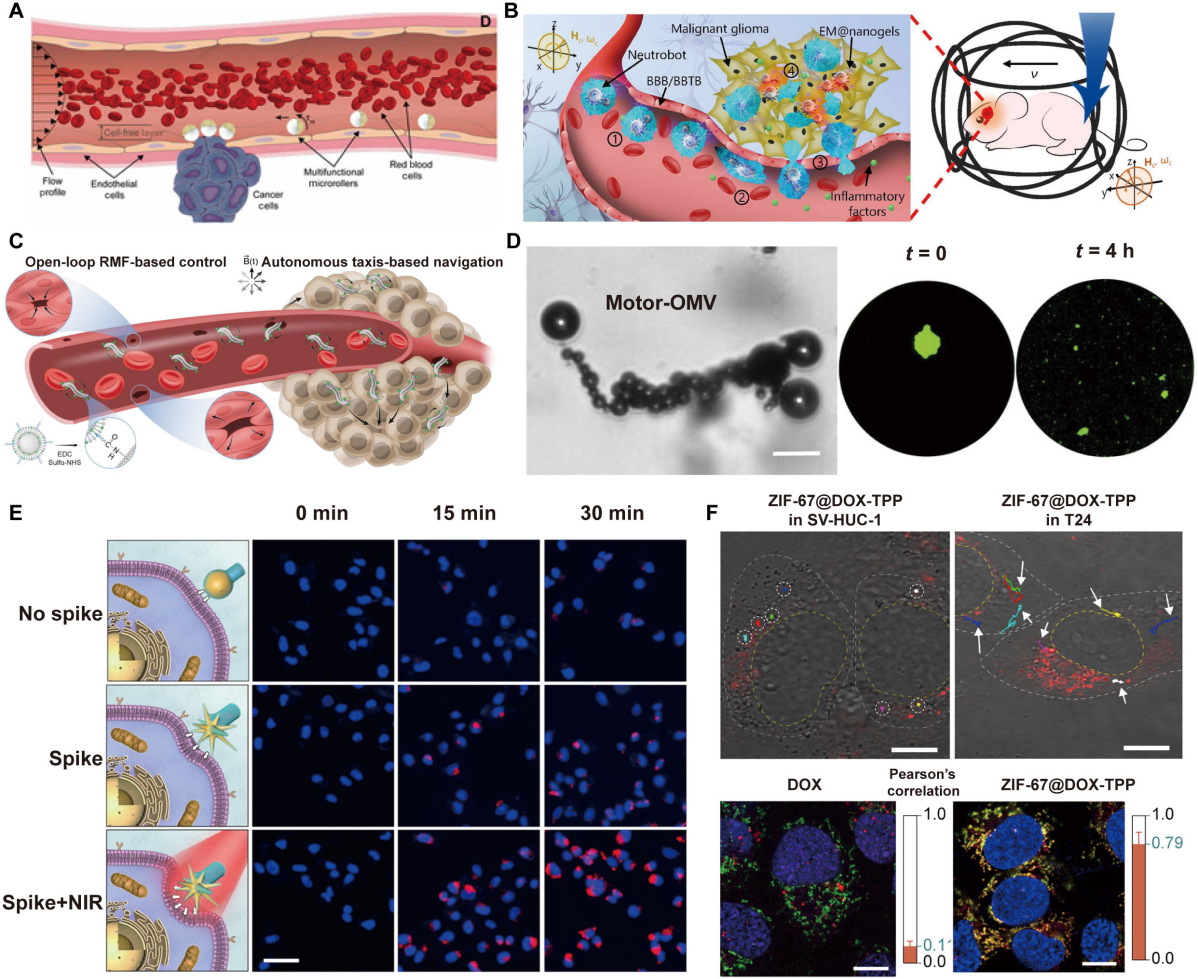
(A) Schematic of magnetically driven microrollers moving upstream against blood flow along the vessel wall [27]. Copyright 2020, American Association for the Advancement of Science. (B) Schematic of active delivery of dual-responsive neutrobots toward malignant glioma [171]. Copyright 2021, American Association for the Advancement of Science. (C) Schematic depicting the magnetically guided transport of MTB–liposome conjugates through a blood vessel, navigating dynamic cell gap openings and targeting a tumor [173]. Copyright 2022, American Association for the Advancement of Science. (D) Optical images depict the paths of uncoated Mg micromotors and Motor-OMV in phosphate-buffered saline (PBS) at a pH of 5. Additionally, fluorescence imaging highlights exemplary tumor cell spheroids expressing GFP (green) prior to and following their exposure to Motor-OMV [174]. Copyright 2021, Wiley-VCH GmbH. (E) Fluorescence images showing tumor cells treated with nanoparticles lacking nanospikes, spiky nanoparticles, and spiky nanorobots at 0, 15, and 30 min post-intervention [178]. Copyright 2024, Springer Nature. (F) Confocal laser scanning microscopy (CLSM) images revealing the intracellular motion paths of ZIF-67@DOX-TPP nanorobots within both tumor cells and human uroepithelial cells. Fluorescence images demonstrate the colocalization of mitochondria in tumor cells following incubation with DOX and ZIF-67@DOX-TPP [68]. Copyright 2023, American Association for the Advancement of Science.

For intravenously administered antitumor drugs to be effective, they must first extravasate the walls of blood vessels and accumulate in the tumor tissue to achieve a sufficient concentration. Alapan et al*.* [27] introduced multifunctional microrobots, inspired by leukocytes and sized similarly to blood cells, for the active delivery of cargo and propulsion under the influence of blood flow (Fig. 6A). It can recognize tumor cells via cell-specific antibodies to achieve targeted drug delivery. The brain’s microenvironment seriously hinders the therapeutic effect of drugs on primary brain tumors and brain metastases [168]. The blood–brain barrier (BBB) is a selective permeability barrier that protects the brain from harmful substances while maintaining a stable environment for neural function. Composed of tightly packed endothelial cells that line the brain’s capillaries, the BBB restricts the passage of large or hydrophilic molecules, allowing only essential nutrients, such as glucose and amino acids, to enter the brain [169]. While the BBB is crucial for maintaining neuronal health, it also poses challenges for drug delivery in treating neurological disorders, as many therapeutic agents struggle to cross this barrier. Understanding the mechanisms that govern BBB permeability is essential for developing effective treatments for conditions like brain tumors. Neutrophils (NEs) possess the ability to traverse the BBB via chemotaxis along inflammatory factor gradients in conditions of inflammation [170]. Leveraging this migratory capability, NEs have been harnessed as drug transporters for precise targeting of inflamed tumor sites. Zhang et al*.* [171] reported a NE-based microrobot (“neutrobot”) that can actively deliver cargoes to malignant glioma in vivo, achieving targeted drug delivery to brain tumors (Fig. 6B).

#### Tumor ECM infiltration

Solid tumors present a significant challenge for effective drug delivery due to several physical and biological barriers. These tumors often exhibit a dense ECM that can impede the diffusion of therapeutic agents [172]. These factors complicate the effective entry of drugs, often leading to suboptimal therapeutic outcomes and highlighting the need for innovative delivery strategies. Gwisai et al*.* [173] presented a magnetic torque-driven control system to improve transport across biological barriers. The bacteria exhibit innate taxis toward tumor cores (Fig. 6C). This study highlights how magnetic torque-driven control methodologies can enhance tumor accumulation. Besides, bubble-propelled microrobots could also be used to breach solid tumor barriers. These tiny devices harness the power of gas bubbles generated through chemical reactions to propel themselves through fluids quickly. Typically constructed from biodegradable materials, bubble-propelled microrobots can navigate complex environments, making them particularly promising for applications in biomedical fields, such as targeted drug delivery and minimally invasive surgery. Zhou et al*.* [174] reported the creation of a microrobot-based strategy for tissue disruption (Fig. 6D). They employed a magnesium-based microrobot system capable of interacting with water to generate a propulsion force within solid tumors, leading to tumor tissue disruption. To assess the damaging impact of microrobots, a 3D tumor spheroid system was created using MC38 cells modified to produce green fluorescent protein (GFP). Imaging of the spheroids using fluorescence showed substantial structural damage following a 4-h exposure to the microrobots.

#### Intracellular delivery

Tumor intracellular delivery is a vital focus in cancer therapy, aimed at effectively transporting therapeutic agents directly into tumor cells to enhance treatment efficacy. Advanced techniques, such as NP-based systems and liposomes, are designed to encapsulate drugs or genetic material and facilitate their uptake by cancer cells [175]. Additionally, researchers are exploring innovative approaches such as stimuli-responsive systems that release their payload in response to the unique microenvironment of tumors, including pH changes [176] or enzyme response [177] By improving intracellular delivery into tumor cells, these strategies aim to potentiate the therapeutic impact and overcome resistance mechanisms, paving the way for more effective cancer treatments. By making some special designs on the morphology of microrobots, it can be facilitated to enter the cell. Yan et al*.* [178] developed biomimetic nanorobots with a head and hollow tail using a site-selective superassembly strategy. Their self-propulsion capability is induced by temperature gradient, and spiky heads significantly enhance cellular uptake at interfaces, facilitate transvascular extravasation, and improve intratumoral penetration. As Fig. 6E shows, spike-robots with NIR irradiation have better cell entrance ability. Upon entering the cell, microrobotic swarms face the risk of degradation due to cellular internalization and subsequent sequestration within lysosomes, where hydrolytic enzymes and acidic conditions prevail [179–181]. Therefore, the release from lysosomes is critical for transporting microrobots into the cytoplasm and for their effective intracellular function. Various strategies have been developed to promote the escape of nanomaterials from lysosomes. Certain positively charged substances function as drug carriers that enter lysosomes, destabilizing the negatively charged lysosomal membrane through electrostatic interactions, which facilitates lysosomal escape [182]. However, positively charged materials are rapidly cleared from circulation, limiting their ability to reach tumors effectively [183]. Alternatively, extracellular membrane vesicles that express fusion peptides can achieve membrane fusion due to conformational changes induced by the acidic environment of lysosomes [184]. Nonetheless, all these strategies primarily depend on passive diffusion to target tumor sites. Peng et al*.* [68] introduced autonomous nanorobots for active drug delivery targeting mitochondria (Fig. 6F). These microrobots are created by simply encapsulating mitochondriotropic doxorubicin-triphenylphosphonium (DOX-TPP) within zeolitic imidazolate framework-67 (ZIF-67) NPs. ZIF-67 can decompose bioavailable H_2_O_2_ to lead to lysosome escape. As Fig. 6F shows, ZIF-67@DOX-TPP nanorobots exhibited effective motion tracking with colored trajectories inside the T24 bladder cancer cell compared to normal cells (SV-HUC-1). From the fluorescent imaging, the Pearson’s correlation coefficient value of the ZIF-67@DOX-TPP group (0.97) was higher than the DOX group (0.11), which means that the ZIF-67@DOX-TPP group possesses better lysosomal escape.

Microrobotic swarms offer significant advantages in drug delivery owing to their collective intelligence and adaptive capabilities. Their coordinated behavior enables enhanced navigational precision, allowing the swarm to dynamically adjust its trajectory in complex biological environments and effectively overcome obstacles. The adaptability facilitates efficient long-range delivery and enables the swarms to penetrate dense tumor tissues. The success of active delivery depends on precise navigation and fast-response control. Therefore, medical imaging technologies enabling control of microrobotic swarms is critical, which will be reviewed and discussed in the following section.

## Imaging of Microrobotic Swarm in Cancer Therapy

In the realm of cancer therapy, innovative imaging techniques play a crucial role in enhancing treatment efficacy and precision. To effectively navigate and control microrobotic swarms, their positions must be monitored in real time, necessitating the use of efficient imaging methods [185].

### Imaging microrobotic swarms in active delivery

In vivo imaging faces several restraints, such as tissue thickness and the effects of blood flow, which hinder the effective observation of the position and movement of individual agents. However, microrobotic swarms can significantly improve imaging contrast due to their high agent concentration. Integrating advanced imaging techniques with microrobot swarm delivery enables real-time tracking and visualization of the swarms’ movements, as well as their interactions with biological tissues and cells. Various imaging techniques have been recently explored to visualize microrobotic swarms, including fluorescence imaging, MRI, ultrasound imaging, and photoacoustic imaging (PAI).

#### Fluorescence imaging

**Fig. 7.**
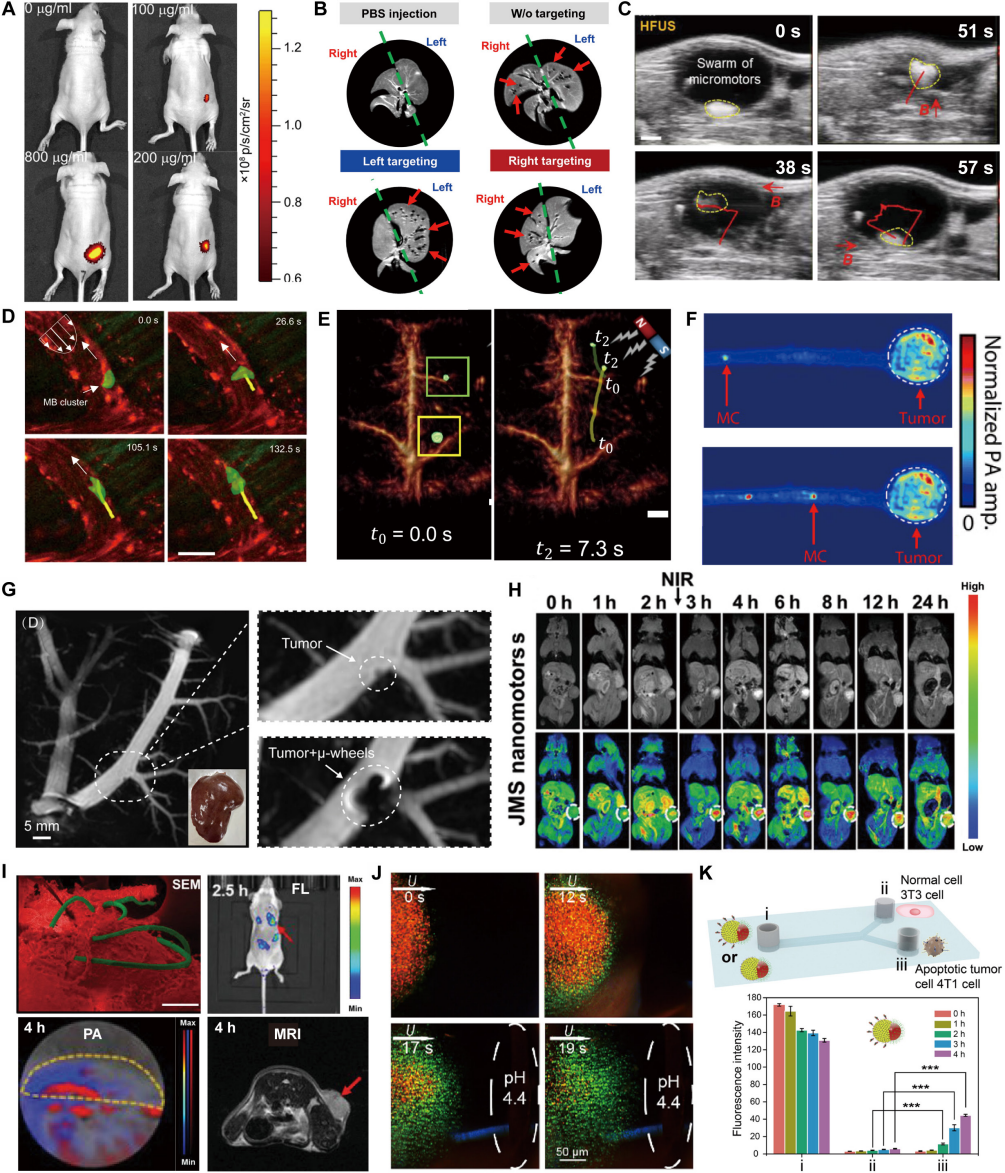
(A) Fluorescence of MSP-72h with varied concentrations in the subcutaneous tissue of nude Balb/c athymic mice [189]. Copyright 2017, American Association for the Advancement of Science. (B) MR images of rat livers following PBS injection, with and without targeting, as well as left and right targeting. Red arrows highlight the microrobots delivered to the liver [192]. Copyright 2022, American Association for the Advancement of Science. (C) High-frequency ultrasound imaging of the swarm of micromotors swimming inside a mouse bladder [195]. Copyright 2021, Wiley-VCH GmbH. (D) The top schematic illustrates microrobotic swarm navigation upstream accompanied by simultaneous growth [200]. Copyright 2023, Springer Nature. (E) Microrobots move inside the blood-filled brain controlled by magnets [204]. Copyright 2022, American Association for the Advancement of Science. (F) Time-lapse PACT images of the migration of an MC toward a model colon tumor [18]. Copyright 2022, American Association for the Advancement of Science. (G) MRI results of a simulated early tumor and tumor plus μ-wheels in the intrahepatic bile duct of a pig [34]. Copyright 2024, American Chemical Society. (H) MRI of mice at different time points before and after injection of JMS NPs. The tumor area is marked by white circles [41]. Copyright 2021, Wiley-VCH GmbH. (I) SEM image of the MSP (green) accumulation in tumor tissue (red). In vivo fluorescence imaging, PA imaging, and MR images of tumor-bearing mice post-intravenous injection of MSP [205]. Copyright 2020, Wiley-VCH GmbH. (J) Time-lapse microscopy captured the dynamic visual pH sensing of a swarm as it collectively transitioned from a microwell with pH 7.4 to a targeted capillary containing a pH 4.4 PBS buffer [206]. Copyright 2023, Wiley-VCH GmbH. (K) Schematic of the Y-shaped microfluidic channel with 3 reservoirs: i, nanomotors; ii, normal cell 3T3 cells; iii, tumor cell 4T1 cells. Variation in fluorescence intensity of nanomotors across various reservoirs over a period of 4 h [210]. Copyright 2021, American Chemical Society.

Fluorescence imaging is a noninvasive technique that enables researchers to visualize biological processes occurring within living organisms [186]. The fluorescent marker is usually a molecule that, when exposed to excitation light of a specific wavelength, absorbs energy and transitions to an excited state, then emits radiation in a short period. This emitted light has a specific wavelength and intensity that can be captured and recorded by specialized fluorescence imaging equipment [187]. Fluorescent imaging provides high sensitivity and molecular specificity, making it feasible for visualizing cellular-level imaging signals. However, its utility is often restricted by limited tissue penetration and photobleaching, which can compromise long-term monitoring in deep tissues [188]. Autofluorescence from microalgae chloroplasts is used to perform fluorescence imaging. The fluorescence signal of the microalgae-based microrobot that lost its motility dropped sharply within 4 h and almost disappeared after 12 h, while the microalgae-based microrobots with motility cleared slowly in the lungs, with 86% and 65% of the fluorescence signal present at 4 and 24 h, respectively, indicating that the motility of the microrobots greatly improved the fluorescence imaging effect [5]. By equipping swarms with fluorescent markers or payloads, researchers can track their movements and interactions in real time within complex biological environments. Yan et al*.* [189] reported helical microrobots equipped with Fe_3_O_4_ and microalgae, developed for in vivo imaging-guided therapy. The intrinsic characteristics of microalgae enable in vivo fluorescence imaging and remote diagnostic sensing, eliminating the need for surface modifications. As shown in Fig. 7A, different concentrations of microrobots were injected subcutaneously into mice. The fluorescence intensity increased with sample concentration in mice.

#### Magnetic resonance imaging

MRI is a medical imaging technique that uses magnetic fields and radio waves to create clear images of organs and tissues in the body. By utilizing MRI’s superior spatial resolution and contrast capabilities, researchers can visualize these swarms’ real-time dynamics and movements. MRI has satisfactory spatial resolution and deep tissue imaging capabilities. However, its extended imaging times lead to low temporal resolution, making the real-time tracking of mobile microrobotic swarms challenging [190]. Microrobots can be wrapped with superparamagnetic NPs to perform T2-weighted MRI on tumor-bearing mice, and the result demonstrated that high T2 signals in deep tumor tissues were detected [191]. The integration of MRI with microrobotic swarm technology enhances the tracking and monitoring of swarm behaviors and facilitates the assessment of their interactions with various biological structures. Go et al*.* [192] depicted microrobots visualized in real-time MRI, facilitating precise delivery of therapeutic and imaging agents to tumor-feeding vessels (Fig. 7B). The MR images illustrated a contrast: Without magnetic guidance, microrobots dispersed throughout the entire liver, yet with magnetic guidance, they predominantly congregated in the targeted liver lobe.

#### Ultrasound imaging

Ultrasound imaging uses high-frequency sound waves to produce images of the inside of the human body. Utilizing ultrasound imaging to visualize and track the movements of microrobotic swarms in real time as they navigate within biological environments is a groundbreaking approach. This noninvasive imaging modality provides high-resolution, dynamic images, allowing for the effective monitoring of swarm behavior and facilitating enhanced understanding of their interactions with target tissues, such as tumors or infected areas [15,17,193]. Ultrasound offers real-time imaging with high compatibility. Its performance, however, can be adversely affected by acoustic interference and artifacts, resulting in reduced resolution and contrast, particularly in complex tissue environments. Hydrogel-based image-guided bioabsorbable acoustic microrobotic swarms could be promising in tackling the problem. Ultrasound was applied to excite and oscillate microbubbles, achieving efficient propulsion and enhanced imaging [194]. By integrating ultrasound imaging with microrobotic swarms, researchers can optimize delivery processes, study therapeutic efficacy, and assess the intricate dynamics of treatment delivery in real time. Aziz et al*.* [195] introduced a controllable movement of the swarm in the bladder of living mice under real-time ultrasound observation (Fig. 7C). This method allows for the discrimination of the microrobots’ signals from those of endogenous chromophores using ultrasound imaging. Furthermore, microbubbles play a crucial role in ultrasound imaging and ultrasound-mediated gene or drug delivery, offering valuable applications within the field [196–198]. Functioning as contrast agents, these microbubbles can provide details about targeted biomarkers or specific cell types at the molecular scale. By injecting these microbubbles into an individual’s bloodstream, their trajectory can be monitored as they traverse the intricate network of small blood vessels responsible for delivering oxygenated blood to the heart muscle [199]. Leveraging microbubbles, imaging techniques can be employed to observe and analyze the intricate swarming behavior displayed by microrobots. Del Campo Fonseca et al*.* [200] introduced lipid-shelled microbubbles that autonomously aggregate and propel under ultrasound irradiation. As depicted in Fig. 7D, the swarms navigated upstream along the vasculature. A swarm, highlighted by a green overlay and an arrow, was observed within a venule. The movement of the swarm was manually traced with a yellow line as it advanced against the flow, accumulating additional bubbles along its trajectory. The combination of ultrasound imaging and microrobotic swarms represents a significant leap forward in minimally invasive interventions.

#### Photoacoustic imaging

PAI represents a cutting-edge approach to medical diagnostics, utilizing the photoacoustic phenomenon. This effect arises when pulsed laser energy is absorbed by biological tissues, causing thermal expansion, pressure changes, and the generation of acoustic waves [201]. This process induces pressure alterations, culminating in the production of detectable sound waves. PAI provides a promising balance between sensitivity and spatial resolution, but challenges remain in achieving sufficient penetration depth, especially in densely structured tissues [202]. A new type of swarm can be prepared with lanthanide-doped upconversion NPs as the core and covalent organic frameworks as the shell. Blue fluorescence was emitted by upconversion NPs, and the detectable multispectral photoacoustic tomography signal was generated [203]. Compared with traditional photoacoustic tomography imaging contrast agents, it has better imaging depth and long-term environmental stability. Wrede et al*.* [204] proposed a noninvasive method for the real-time detection and tracking of circulating microrobotic swarms using PAI. As illustrated in Fig. 7E, a magnet was employed to manipulate the 5-μm-diameter microrobots within a blood-filled brain. After 11.5 s of manipulation, 2 clusters in the right periphery were observed moving toward the magnet, propelled by the induced magnetic force. Photoacoustic computed tomography (PACT) is an imaging technique that synergizes the optical contrast benefits of traditional optical imaging, which enhances the richness of imaging data, with the acoustic resolution advantages of conventional ultrasound imaging. This combination enables the acquisition and computer-assisted reconstruction of images of biological tissues. PACT is characterized by high spatiotemporal resolution and significant tissue penetration. Wu et al*.* [18] conducted a study utilizing PACT to monitor the behavior of microrobotic swarms in vivo within the intestines. As illustrated in Fig. 7F, the swarms effectively navigated toward the targeted colon tumor after injecting into the intestines.

### Imaging microrobotic swarms enriched within tumor tissue

Once microrobotic swarms arrive at the tumor site, they can serve as contrast agents for tumor imaging and sensing.

#### Tumor imaging

Microrobotic swarms can navigate complex biological environments, delivering imaging modalities such as fluorescence or ultrasound to enhance the detection and characterization of tumors with high resolution. Zhou et al*.* [34] introduced a novel approach utilizing magnetic microwheels (μ-wheels) for tumor detection by presetting the direction of the RMF. The mechanism that allows μ-wheels to locate tumors autonomously is based on a wall-guided self-navigation strategy inspired by intracellular motor proteins. The μ-wheels are propelled by an external RMF, which is preset with an initial angle and remains constant during navigation. As the μ-wheels roll along the inner walls of microtubes, their motion is guided by hydrodynamic interactions with the walls. When the μ-wheels encounter a tumor, the flow field around them is disrupted due to the presence of the tumor. This disruption breaks the symmetry of the rotational flows, causing the μ-wheels to lose their ability to move along the walls, and accumulate and aggregate around the tumor site. As Fig. 7G shows, pinpointing minute tumors remains a complex task despite employing the most advanced resolution techniques. Nonetheless, the μ-wheels can accurately locate tumors and improve MRI accuracy, enabling early diagnosis of tumors. Zheng et al*.* [41] proposed another method to enhance MRI for tumor detection. They engineered a Janus mesoporous silica nanomotor (JMS nanomotor) that responds to NIR light, advancing MRI capabilities in live organisms. As depicted in Fig. 7H, when subjected to NIR light, the tumor’s MR signal demonstrated remarkable enhancement across all observed time intervals compared to the control group without NIR laser stimulation.

Each imaging technique offers distinct advantages, yet they also come with significant limitations that must be considered. For instance, fluorescence imaging typically has a limited depth of penetration, and certain dyes may lack biocompatibility. Consequently, integrating multiple imaging modalities can enhance overall imaging capabilities, leading to more accurate diagnostics and improved treatment outcomes. Zhong et al*.* [205] proposed a photosynthetic biological hybrid microrobot system composed of platensis and Fe_3_O_4_. Platensis is naturally fluorescent and rich in chlorophyll, which enables it to have the ability of fluorescence and PAI, and the superparamagnetic Fe_3_O_4_ coating can improve the ability of MRI. Figure 7I indicates that microrobots can target tumors and perform multiple types of detection, including fluorescent imaging, PAI, and MRI.

#### Sensing

The collective behavior of microrobotic swarms can amplify their effectiveness in mapping out tumor locations, characterizing tumor properties, and transmitting this vital information in real time to medical professionals for prompt analysis and decision-making. The microrobotic swarms can adapt to the TME, providing real-time data while minimizing invasiveness. Yu et al*.* [206] demonstrated that swarming magnetic photonic-crystal microrobots (PC-bots) can detect on-the-fly visual pH spontaneously. As Fig. 7J shows, combining magnetic actuation and pH-regulated drug loading/release, this innovative swarm of magnetically driven microrobots functions as an intelligent diagnostic and therapeutic platform, spontaneously performing dynamic visual pH detection and self-regulating drug delivery by responding to local pH signals. Besides, DNA is typically released only by aging and apoptotic cells. Elevated concentrations of DNA indicate an abnormal cellular state [207,208]. Consequently, cell-free tumor DNA released from dying or apoptotic tumor cells has potential as a biomarker for early diagnosis, prognosis, and monitoring [209]. Ye et al*.* [210] introduced a deoxyribonuclease-functionalized Janus NP (JNP) nanomotor system, which can be activated by DNA concentrations as low as nanomolar to micromolar levels (Fig. 7K). These cargo-loaded nanomotors can detect DNA signals released by cells, exhibiting directional movement toward tumor cells. A schematic illustration depicts a Y-shaped microfluidic channel containing nanomotors in reservoir i, normal 3T3 cells in reservoir ii, and tumor 4T1 cells in reservoir iii. Data show that after 4 h of incubation, the fluorescence intensity in the tumor cells is significantly higher than that in the normal cells. These innovative approaches not only improve diagnostic accuracy and monitoring of tumor progression but also hold promise for future therapeutic applications, enabling targeted drug delivery directly to cancer cells.

Microrobotic swarms have advantages in providing enhanced imaging for cancer. The swarming behavior allows the microrobots to navigate and accumulate in targeted regions, enhancing local imaging contrast. Additionally, microrobots can be functionalized with various imaging agents, making the swarms highly versatile platforms for multimodal imaging. This synergy between microrobotic swarms and advanced imaging techniques holds the potential to overcome conventional imaging challenges, offering more precise, responsive, and integrated diagnostic capabilities in complex biological environments. The therapeutic impact on cancer treatment will be reviewed in the next section.

## Microrobotic Swarms Enabled Cancer Therapy

Microrobotic swarms have brought new hope to cancer therapy [211]. The utilization of microrobotic swarms for the delivery of therapeutic agents, including cells and NPs, has emerged as a promising strategy in tumor treatment. Compared to conventional therapies, microrobotic swarms address critical issues of high dosage requirements, off-target distribution, and poor retention. However, significant challenges remain, including limited drug-carrying capacity and underdeveloped in vivo imaging techniques [212]. Consequently, the clinical translation of microrobotic swarms still faces considerable hurdles and requires further advancements. Cancer can be classified based on location, including brain, lung, liver, GI, and bladder cancer. Cancers occurring in different organs possess distinct biological characteristics, and the corresponding treatment approaches vary accordingly. We summarize the typical work of microrobotic swarms for cancer therapy of different organs (Table).

**Table.** The material, propulsion mechanism, agent design, imaging method, and treatment of some typical microrobotic swarms for cancer therapy of different organs.

Type of cancer	Material	Propulsion mechanism	Agent design	Imaging method	Treatment	Ref.
Lung cancer	Spirulina platensis	/	Gold nanoclusters	Fluorescence	Radiotherapy	[217]
	Microalgae	/	DOX	Fluorescence	Chemotherapy	[164]
	Nanogel	Chemical fuels	DOX	Fluorescence	Chemotherapy	[257]
	Nanoemulsion	Chemical fuels	Osimertinib	MRI	Chemotherapy	[258]
Brain cancer	Neutrophils	Magnetic field	Paclitaxel	Fluorescence	Chemotherapy	[171]
	Graphene	Magnetic field	Epirubicin	Fluorescence	Magnetic hyperthermia and chemotherapy	[221]
	Natural killer cell membrane	Optical field	Aggregation-induced emission	Fluorescence	Photothermal	[222]
	Zwitterionic polymer	Chemical fuels	Triphenylphosphine	Fluorescence	Immunotherapy	[224]
Liver cancer	Hydrogel-enveloped porous structure and magnetic nanoparticles	Magnetic field	DOX and fluorouracil	X-ray and MRI	Chemotherapy	[192]
	Fe_3_O_4_ and SiO_2_	Magnetic field	DOX	/	Chemotherapy	[227]
	Pd@ZIF-8	Optical field	Resiquimod	Fluorescence	Immunotherapy	[231]
Bladder cancer	293T cells	Magnetic field	cRGD tumor targeting peptide	Fluorescence	Oncolytic viral therapy	[8]
	Bacterial outer membrane vesicle	Chemical fuels	siRNA	Fluorescence	Gene therapy	[104]
	Bacterial outer membrane vesicles	Magnetic field	Macrophages, antitumor peptides of OMVs	Fluorescence	Immunotherapy	[143]
Gastrointestinal cancers	Phage	Chemical fuels	Irinotecan	Fluorescence	Chemotherapy	[240]
	Mitochondrial N770-conjugated mesoporous silica nanoparticles	Chemical fuels	CaO_2_	Fluorescence	Hyperthermia	[241]
	Lipid-protamine-DNA nanoparticle	Chemical fuels	An engineered LPS-targeting fusion protein	Fluorescence	Immunotherapy	[259]
Breast cancer	Spirulina platensis	Magnetic field	Spirulina platensis	Photoacoustic imaging and MR imaging	PDT	[205]
	Cisplatin-skeletal zeolitic imidazolate frameworks	Chemical fuels	Reactive oxygen species	Fluorescence	Synergetic chemotherapy	[126]
	Macrophages	Magnetic field	DOX	Fluorescence	Chemotherapy	[165]
	AuNS	Optical field	DOX	Fluorescence	Chemotherapy	[178]
	Zeolitic imidazolate framework-67	Chemical fuels	DOX-TPP	Fluorescence	Immunotherapy	[68]
Prostate cancer	Poly calcium phosphate hybrid nanomotor	Chemical fuels	ΑPD-L1 antibody	Fluorescence	Immunotherapy	[118]

### Lung cancer

**Fig. 8.**
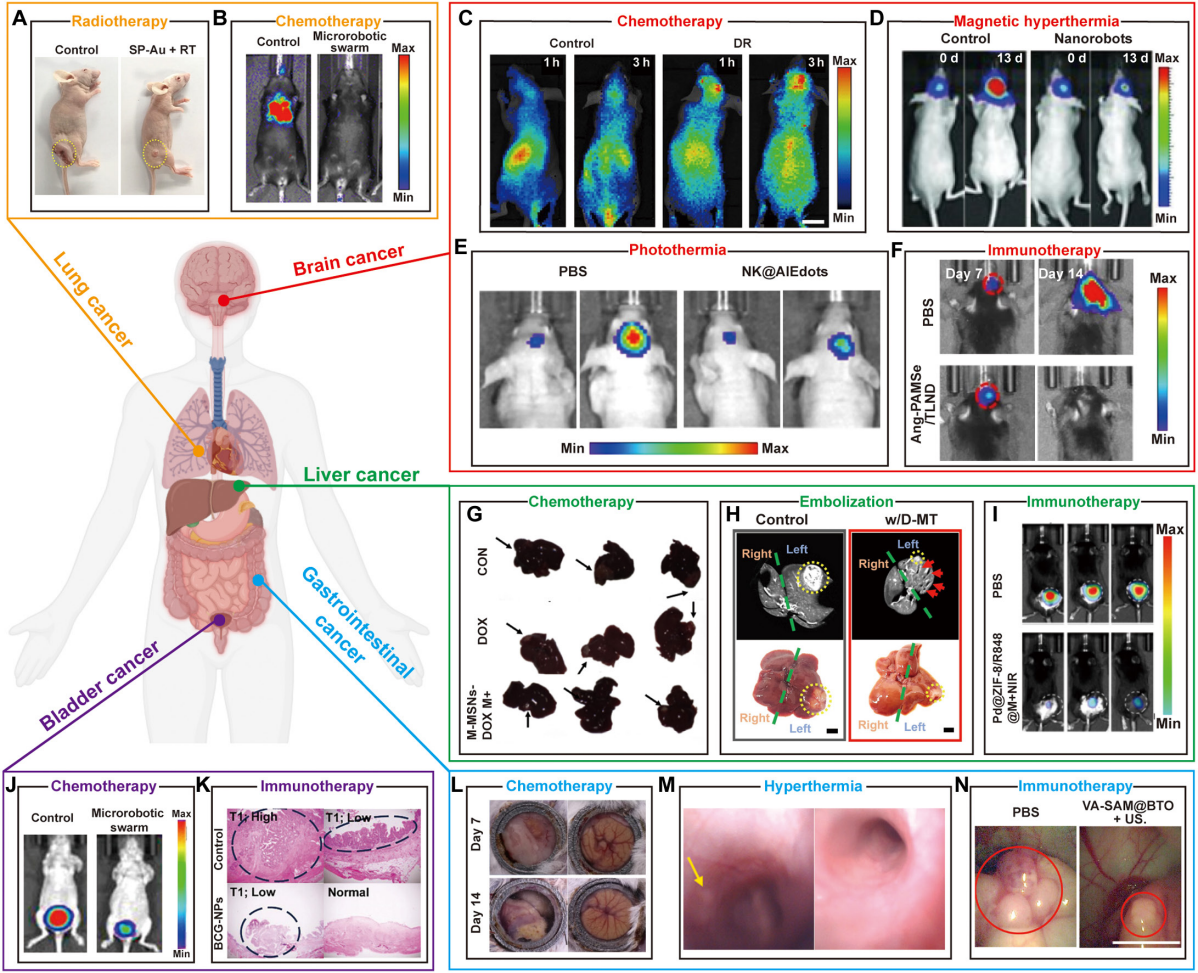
Microrobotic swarms for cancer therapy. (A) Representative photographs of lung cancer subcutaneous tumor model at day 10 after SP-Au with radiotherapy [217]. Copyright 2024, Springer Nature. (B) Bioluminescence images of mice treated with PBS, or microrobotic swarm after treatment [164]. Copyright 2024, American Association for the Advancement of Science. (C) Fluorescence imaging conducted in vivo on mice with gliomas included a control group as well as a group subjected to surgical intervention and exposed to a magnetic field for 1 h (DR group) [171]. Copyright 2021, American Association for the Advancement of Science. (D) Optical luminescence imaging in vivo of luminescence-labeled U87 tumors xenografted into nude mice brains and treated without (control) or with nanorobots and low-frequency ultrasound for 13 d [221]. Copyright 2013, Wiley-VCH GmbH. (E) Representative in vivo bioluminescence images of orthotopic glioma at different time points after PTT [222]. Copyright 2020, American Chemical Society. (F) Bioluminescence imaging was conducted on the PBS group and the Ang-PAMSe/TLND group, with red circles highlighting the chemiluminescence imaging. Images were taken on the 7th and 14th days following the treatment [224]. Copyright 2023, Springer Nature. (G) Photographs comparing saline-treated mice to those treated with free DOX and M-MSNs DOX under the influence of a magnetic field [227]. Copyright 2016, Elsevier Ltd. (H) MR and optical images of the livers of rats in the control and with DOX-loaded microrobot targeting (w/D-MT) groups. Red arrows on MR images indicate microrobots delivered to the rat liver. Yellow circles indicate liver tumors in rats [192]. Copyright 2022, American Association for the Advancement of Science. (I) Luminescence images of mice injected with PBS and Pd@ZIF-8/R848@M+NIR for tracking the growth of Hepa1–6-luc tumors [231]. Copyright 2024, American Chemical Society. (J) Bioluminescence images captured in vivo of T24 tumor-bearing mice following hydrogen chemotherapy with different treatment regimens: PBS and microrobotic swarm combined with laser [234]. Copyright 2020, American Chemical Society. (K) Bladder tissue staining of rats from various groups, including the control and BCG-NPs groups. The tumor regions are delineated with a black dotted line [236]. Copyright 2024, Wiley-VCH GmbH. (L) An abdominal imaging window was surgically implanted into the mice for long-term observation of therapeutic effects. The windows were placed prior to treatment, with the PBS group on the left and the A-phage and D-IDNP group on the right. The images shown are representative of 3 biological replicates. The tumor mass appeared grayish white [240]. Copyright 2019, Springer Nature. (M) Endoscopic images of the colons from various mouse groups: control group (left) and CaO_2_-N770@MSNs (+ NIR + αPD-L1) group (right) [241]. Copyright 2023, Elsevier Ltd. (N) Laparoscopic images of orthotopic CRC mice on day 10 [242]. Copyright 2024, American Association for the Advancement of Science.

Lung cancer is the leading cause of cancer-related deaths worldwide [213]. The current treatment strategies for lung cancer remain confined to clinical approaches such as surgical resection, radiotherapy, and chemotherapy. However, these methods are characterized by high invasiveness or lack of specificity and are often associated with severe adverse effects due to their significant toxicity to normal cells and tissues. The lungs present unique challenges for microrobotic swarms due to their complex, branching bronchial pathways, dynamic airflow, and mucus-rich environment [214]. To effectively target lung tumors, microrobots must be designed with aerodynamic architectures to navigate narrow airways, and mucoadhesive coatings to resist mucociliary clearance [215]. Chen et al*.* [216] proposed a magnetic microrobot swarm composed of microgel particles for intrabronchial targeted delivery. Magnetic propulsion systems allow precise navigation through bronchial pathways, and pH-sensitive drug carriers ensure localized release in acidic TMEs. Consequently, the development of precision medicine has become imperative. Recent advancements have demonstrated promising progress in integrating microrobots with chemotherapy agents to enhance therapeutic outcomes. Hua et al*.* [217] reported an Au-based micromotor to enhance radiotherapy for lung cancer, as shown in Fig. 8A. After 10 d of treatment, the tumor size was significantly suppressed. With rising concentrations, the efficacy in annihilating tumor cells improves, suggesting that the tri-bead microrobots hold significant promise for precise chemo-PTT to treat lung cancer cells. In addition, Zhang et al*.* [164] used mobile algae to tackle lung metastasis by delivering drug-carrying NPs precisely. The microalgae autonomously propel within the lungs, enabling regulated drug discharge and amplified drug scattering to combat metastasis effectively. Through intratracheal infusion, the algae-NP(DOX)-robots effectively ferry their medication cargo into the lung depths, all the while sustaining uninterrupted motion, resulting in swift drug propagation, heightened tissue accumulation, and prolonged drug residence, surpassing passive drug-laden NPs and free drug counterparts in efficacy (Fig. 8B).

### Brain cancer

Brain cancer remains among the deadliest forms of cancer, with their mortality rate remaining virtually unchanged over the past 30 years. Overcoming the BBB has consistently been a major challenge in the treatment of brain cancers [218]. Microrobotic swarms targeting brain tumors must overcome the BBB and navigate narrow, tortuous cerebral vasculature. Design strategies include ultra-small sizes (<200 nm) to enable BBB penetration, magnetic or acoustic propulsion for steering, and surface functionalization with ligands (e.g., transferrin) to exploit receptor-mediated transcytosis [168,219]. Localized hyperthermia or enzymatic triggers can enhance drug release at the tumor site. Zhang et al. [171] utilized natural NEs to phagocytose drug-loaded magnetic nanogels encapsulated with *E. coli* membranes to create a NE-based microrobot (“neutrobot”). This robot features dual responsiveness to magnetic fields and chemical signals, allowing it to precisely navigate to malignant gliomas, penetrate the BBB, and release chemotherapy drugs within the tumor for targeted treatment (Fig. 8C). Hyperthermia is a therapy technique in which the pathological tissue is exposed to high temperatures [220]. Solely specific regions proximate to microrobotic swarms experience temperature elevation beyond the body’s usual range. Consequently, this approach offers the potential to eradicate cancerous cells while mitigating adverse impacts on healthy tissue. Yang et al*.* [221] introduced a nanoscale magnetic graphene oxide (GO) drug carrier for epirubicin delivery. GOs can function as an effective heat-conducting substrate, enabling localized temperature increase when exposed to low-power focused ultrasound (FUS), facilitating deep-seated hyperthermia and drug delivery (Fig. 8D). PTT is another method of hyperthermia, where light energy can be transformed into heat to raise the temperature by using light-absorbing materials. Deng et al. [222] developed AIE nanorobots that simulate natural killer (NK) cells, which can effectively cross the BBB and perform targeted diagnosis and treatment of brain tumors. Under NIR light irradiation, heat can be generated to effectively inhibit tumor growth (Fig. 8E). Previously, researchers believed that the brain was an immune privileged part of the human body. As research progressed, it was discovered that the brain could communicate with the immune system through cerebrospinal fluid and lymphatic vessels [223]. Chen et al. [224] constructed nitric oxide (NO)-driven zwitterionic polymer-based nanorobots. The elevated NO concentration generated during the chemotaxis phase of nanomotor operation serves as an inducer for immunogenic cell death (ICD), amplifying the synthesis of immune antigens specific to tumors. Consequently, this process supports the maturation of antigen-presenting cells and the stimulation of T cell activation, contributing to enhanced immune responses against cancer cells (Fig. 8F).

### Liver cancer

Liver cancer is also a common type of cancer, with many patients dying from it each year [35]. Traditional chemotherapy drugs, administered systemically, often cause severe complications in the treatment of liver cancer. Liver cancer presents unique challenges for microrobotic swarm-based therapy due to its dense vasculature and strong immune defenses [225]. To navigate the intricate hepatic vasculature, microrobots can leverage magnetic and acoustic actuation for precise intrahepatic targeting and enhance penetration into tumor regions. Additionally, shape-adaptive microrobots capable of transitioning between dispersed and aggregated states can enhance maneuverability and tumor penetration. To evade rapid clearance by the liver’s immune system, microrobots should be coated with biomimetic materials, such as hepatocyte-derived or platelet-membrane coatings, or functionalized with polyethylene glycol (PEG) to prolong circulation time [226]. Shao et al*.* [227] developed versatile Janus nanocomposites featuring a magnetic Fe_3_O_4_ head and a mesoporous SiO_2_ body loaded with DOX acting as “nano-bullets”. By utilizing magnetic field manipulation for tumor site localization and pH-triggered DOX release, significant tumor inhibition efficacy was demonstrated across subcutaneous and orthotopic liver tumor models in murine subjects (Fig. 8G). Transarterial chemoembolization (TACE) is an interventional cancer treatment technique that precisely delivers therapeutic agents to the tumor’s blood supply artery via a catheter while simultaneously blocking the nutrient source. This induces tumor cell death through intense starvation and cytotoxic effects [228–230]. Based on this, Go et al*.* [192] proposed a versatile medical microrobotic platform for live and post-surgery imaging essential for liver chemoembolization. This system facilitates precise targeting, vessel embolization, and controlled drug administration. Their study involved in vivo assessments to gauge the microrobots’ efficacy in a liver tumor scenario (Fig. 8H). The liver tissue is rich in immune cells (macrophages, NK cells, and T lymphocytes), providing a significant natural advantage for the development of immunotherapy for liver cancer. However, due to the influence of the tumor’s immunosuppressive microenvironment, immune cells cannot function normally to fight against tumor cells. Chen et al*.* [231] developed asymmetric nanorobots (Pd@ZIF-8/R848@M JNMs) to enhance photoimmunotherapy of hepatocellular carcinoma. Under NIR-II irradiation, Pd@ZIF-8/R848@M JNMs convert light energy into heat energy, which can penetrate deeper into tumor tissue. At the same time, the immune activator resiquimod (R848) was used to transform the immunosuppressive microenvironment into an immune-activated state, achieving immunotherapy (Fig. 8I).

### Bladder cancer

Bladder cancer is the 4th and 11th most common cancer in men and women, respectively [232]. Under conventional chemotherapy, recurrence is common due to factors such as low drug penetration at the tumor site, drug dilution in urine, and tumor cell drug resistance [233]. By utilizing small, autonomous robots that can navigate the bladder with high accuracy, these swarms offer a minimally invasive way to target and treat cancerous tumors. For bladder cancer, microrobots must adhere to the urothelium and resist urine flow. Buoyant designs (e.g., gas-generating microrobots) improve retention, while stimuli-responsive materials trigger drug release upon contact with the bladder wall [194]. Surface functionalization with lectins or antibodies enhances tumor-specific binding [46]. Swarms should be degradable into urine-soluble components to prevent residual accumulation. To enhance the efficacy of cancer treatment, Sun et al. [234] synthesized fluorine-containing polymers that self-assemble into NPs with peptides, photosensitizers, or sonosensitizers. Under 660-nm laser irradiation, these NPs efficiently produce H_2_ gas in situ, significantly improving the efficacy of hydrogen therapy for tumors both in vitro and in vivo (Fig. 8J). Intravesical Bacillus Calmette-Guérin (BCG) therapy is a well-established approach for treating high-risk nonmuscle invasive bladder cancer [235]. However, more than half of the patients continue to face recurrence or disease progression. Liu et al*.* [236] utilized a “biotin-avidin strategy” to incorporate DOX-loaded NPs into live BCG (DOX@BCG) to improve therapeutic efficacy. BCG’s adhesion to the bladder epithelium facilitates the precise targeting of DOX@BCG to local tumor cells while enhancing drug transport within the tumor. The synergistic effect of BCG immunotherapy and DOX chemotherapy effectively inhibit tumor progression (Fig. 8K). The bladder’s anatomical structure, with its fluid-filled cavity and smooth muscle walls, provides an ideal environment for microrobots to travel through, target tumors, and deliver localized treatments. The proximity of the bladder to the urethra allows for less invasive treatment, minimizing the risk of complications and offering the potential for repeated precise interventions. With the ability to work within this confined space, microrobotic swarms can achieve high levels of precision in targeting cancer cells, offering a more effective and less damaging alternative to traditional treatments like surgery or systemic chemotherapy.

### GI cancer

Colorectal cancer accounts for approximately 10% of newly diagnosed malignancies and cancer-related deaths each year. Patients with metastatic colorectal cancer have a poor prognosis, and there is an urgent need to develop more effective treatment strategies [237]. The acidic environment, peristaltic movements, and mucosal barriers of the GI tract require robust and adaptable microrobotic swarms [238]. Microrobots for GI cancers demand robustness against peristalsis, acidic/alkaline conditions, and enzymatic degradation. Meanwhile, microrobots can use pH-responsive materials for site-specific drug release and asymmetric shapes (e.g., Janus particles) for effective propulsion in luminal fluids. Mucopenetrating coatings (e.g., PEG) enhance tumor access, while magnetic or chemical gradients enable swarm navigation [239]. Biocompatible, nontoxic materials (e.g., silica) are critical to avoid mucosal irritation. The flexibility and maneuverability of microrobotic swarms enable them to navigate the winding and narrow passages of the GI tract, reaching tumors that might otherwise be difficult to treat with traditional methods. Zheng et al. [240] invented a phage-guided bio-hybrid nanomaterial that can modulate the gut microbiota in a mouse model of colorectal cancer, enhancing their response to chemotherapy (Fig. 8L). Additionally, Wu et al. [241] designed an innovative antitumor platform using mitochondria-targeting N770-conjugated mesoporous silica NPs loaded with CaO_2_ (CaO_2_-N770@MSNs). Under NIR irradiation, the microrobots can produce O_2_ and perform hyperthermia, which helps in killing deep-seated tumors. LPS is abundant in primary colorectal cancer tissues and acts as an immunostimulatory ligand that promotes colorectal cancer metastasis (Fig. 8M). Fan et al*.* [242] developed microrobots based on Veillonella (VA) to alleviate the immunosuppressive microenvironment caused by high lactate levels for tumor treatments. As shown in Fig. 8N, the treatment outcome was satisfactory, as the tumor size was significantly suppressed. The coordination and shape-reconfiguration abilities of microrobotic swarms allow them to adapt to the dynamic environment of the GI tract.

## Summary and Outlook

Over the past decade, significant advancements have been made in microrobotic swarms for cancer therapy. This review summarizes recent developments in the design of microrobotic agents, target delivery systems, medical imaging, and their applications in cancer treatment. Despite considerable efforts directed toward in vivo cancer therapy, numerous challenges remain, highlighting the need for further progress in controlling microrobotic swarms to enhance effective targeted delivery and improve biocompatibility.

The application of microrobotic swarms in tumor treatment has been verified in small animal models. However, the devices and control strategies for the actuation of swarms in human-sized areas should be further developed. To achieve the translation from laboratory to clinic, the biocompatibility and toxicity of microrobotic swarms should be thoroughly evaluated. Additionally, integrating swarm agents with natural biological materials may reduce their cytotoxicity. While swarm actuation and control strategies can significantly enhance the targeted delivery of drugs, some agents may still be sequestered by organs and remain within blood vessels during their locomotion [243]. Ensuring that these strategies are harmless to human health remains a significant challenge. Utilizing machine learning and artificial intelligence (AI) algorithms allows swarms to adapt their behavior based on real-time feedback from their environment [244,245]. These algorithms can optimize navigation and improve the swarm’s ability to target specific tissues or tumors. Besides, implementing in vivo imaging techniques (e.g., fluorescence or MRI) allows real-time tracking of swarm behavior and effectiveness, providing insights that can be used to dynamically adjust swarm strategies during treatment.

The clinical translation of microrobotic swarms depends on resolving biocompatibility challenges arising from the interaction with biological systems. First, material toxicity remains a primary concern, as nondegradable components (e.g., metallic NPs) may leach harmful ions or induce oxidative stress [246]. To address this, biodegradable polymers such as polylactic acid (PLA) and polyglycolic acid (PGA) are increasingly employed, as they hydrolyze into nontoxic lactic and glycolic acids [247]. Second, microrobots are easily eliminated by the body’s immune system. Immune recognition poses a barrier to prolonged circulation. Surface modification with PEG or “self” markers like CD47 can effectively reduce macrophage uptake [248]. Third, off-target effects caused by imprecise navigation may damage healthy tissues. Combining magnetic steering swarms with tumor-specific ligands like folate has higher accumulation in tumors [93]. Finally, inappropriate post-therapeutic clearance can result in systemic toxicity. Size-tunable microrobots (<6 nm hydrodynamic diameter) enable renal excretion [249], while iron oxide NPs, widely used for magnetic propulsion, degrade into ferrous/ferric ions that integrate into the body’s iron pool, minimizing systemic toxicity [250]. Through integrated material innovation, surface engineering, and precision control, biocompatibility challenges can be systematically addressed.

The TME presents significant barriers to microrobotic swarm navigation, including dense ECMs, irregular vasculature, and immune response, which often hinder the movement and penetration of microrobotic swarms [115]. To enhance swarm infiltration, microrobotic designs should focus on multifunctionality, combining various propulsion mechanisms to enhance navigational flexibility and payload delivery. Hybrid systems that integrate autonomous and externally controlled actuation will improve adaptability and precision in complex environments. Modifying bacteria with magnetized particles to leverage their natural tumor tropism can enhance tumor infiltration [251].

Controlling microrobotic swarms, especially in dynamic and heterogeneous environments like tumors, demands carefully tailored strategies. Existing strategies encounter critical challenges in achieving high control stability and precision in vivo. Machine learning algorithms have emerged as a powerful tool for enhancing swarm navigation and control [252]. It enables agents to learn optimal policies through iterative interactions with dynamic environments, thus adapting to unforeseen obstacles and nonlinear dynamics typical of in vivo applications [253]. Microrobotic swarms can thus generate autonomous and adaptive control schemes to improve both short-range and long-range delivery capabilities. For example, multi-agent reinforcement learning frameworks allow individual microrobots in a swarm to collaboratively learn from their shared experiences, leading to enhanced collective decision-making and robust performance even in complex and unpredictable scenarios [254]. Moreover, hybrid control architectures that merge machine learning with traditional control algorithms have been investigated [255,256]. These innovative approaches contribute to increased navigational precision, high resilience against environmental disturbances, and improved overall therapeutic efficacy in cancer treatment applications.

Establishing clear ethical guidelines and regulatory frameworks is essential for the clinical translation of microrobotic swarms. Future research should focus on addressing the regulatory hurdles involved in obtaining approval for microrobotic systems, particularly their safety, efficacy, and manufacturing standards. Additionally, comprehensive preclinical and clinical trial designs are needed to evaluate the long-term effects of microrobots in vivo.

Microrobotic swarms hold tremendous potential for revolutionizing cancer treatment by enabling precise, targeted drug delivery and real-time therapeutic monitoring. However, to realize their full clinical potential, continued research is needed to address the existing challenges in design, control, safety, and translation. By focusing on innovative solutions such as hybrid microrobots, AI-driven control systems, multimodal imaging, and personalized therapies, the field can make significant strides toward the clinical implementation of microrobotic swarms in cancer therapy. This will require interdisciplinary collaboration across robotics, materials science, AI, and clinical oncology to overcome the hurdles and bring this promising technology to the forefront of cancer treatment.
